# APLF facilitates interstrand DNA crosslink repair and replication fork protection to confer cisplatin resistance

**DOI:** 10.1093/nar/gkae211

**Published:** 2024-03-23

**Authors:** Cheng-Kuei Wu, Jia-Lin Shiu, Chao-Liang Wu, Chi-Feng Hung, Yen-Chih Ho, Yen-Tzu Chen, Sheng-Yung Tung, Cheng-Fa Yeh, Che-Hung Shen, Hungjiun Liaw, Wen-Pin Su

**Affiliations:** Institute of Clinical Medicine, College of Medicine, National Cheng Kung University, No. 35, Xiao-Tong Road, Tainan 704, Taiwan; Department of Life Sciences, National Cheng Kung University, No. 1 University Road, Tainan City701, Taiwan; Ditmanson Medical Foundation Chia-Yi Christian Hospital, Chiayi City, Taiwan; Ditmanson Medical Foundation Chia-Yi Christian Hospital, Chiayi City, Taiwan; Department of Life Sciences, National Cheng Kung University, No. 1 University Road, Tainan City701, Taiwan; Department of Public Health & Institute of Environmental and Occupational Health Sciences, College of Public Health, National Taiwan University, Taiwan; Institute of Clinical Medicine, College of Medicine, National Cheng Kung University, No. 35, Xiao-Tong Road, Tainan 704, Taiwan; Department of Urology, An Nan Hospital, China Medical University, Tainan, Taiwan; Institute of Clinical Medicine, College of Medicine, National Cheng Kung University, No. 35, Xiao-Tong Road, Tainan 704, Taiwan; Department of Internal Medicine, Chi Mei Medical Center, Tainan, Taiwan; National Institute of Cancer Research, National Health Research Institutes, Tainan 704, Taiwan; Department of Life Sciences, National Cheng Kung University, No. 1 University Road, Tainan City701, Taiwan; Institute of Clinical Medicine, College of Medicine, National Cheng Kung University, No. 35, Xiao-Tong Road, Tainan 704, Taiwan; Departments of Oncology and Internal Medicine, National Cheng Kung University Hospital, College of Medicine, National Cheng Kung University, Tainan 704, Taiwan; Clinical Medicine Research Center, National Cheng Kung University Hospital, College of Medicine, National Cheng Kung University, Tainan 704, Taiwan; Center of Applied Nanomedicine, National Cheng Kung University, Tainan 701, Taiwan

## Abstract

Replication stress converts the stalled forks into reversed forks, which is an important protection mechanism to prevent fork degradation and collapse into poisonous DNA double-strand breaks (DSBs). Paradoxically, the mechanism also acts in cancer cells to contribute to chemoresistance against various DNA-damaging agents. PARP1 binds to and is activated by stalled forks to facilitate fork reversal. Aprataxin and polynucleotide kinase/phosphatase-like factor (APLF) binds to PARP1 through the poly(ADP-ribose) zinc finger (PBZ) domain and is known to be involved in non-homologous end joining (NHEJ). Here, we identify a novel function of APLF involved in interstrand DNA crosslink (ICL) repair and fork protection. We demonstrate that PARP1 activity facilitates the APLF recruitment to stalled forks, enabling the FANCD2 recruitment to stalled forks. The depletion of APLF sensitizes cells to cisplatin, impairs ICL repair, reduces the FANCD2 recruitment to stalled forks, and results in nascent DNA degradation by MRE11 nucleases. Additionally, cisplatin-resistant cancer cells show high levels of APLF and homologous recombination-related gene expression. The depletion of APLF sensitizes cells to cisplatin and results in fork instability. Our results reveal the novel function of APLF to facilitate ICL repair and fork protection, thereby contributing to cisplatin-resistant phenotypes of cancer cells.

## Graphical Abstract

**Graphical Abstract:**
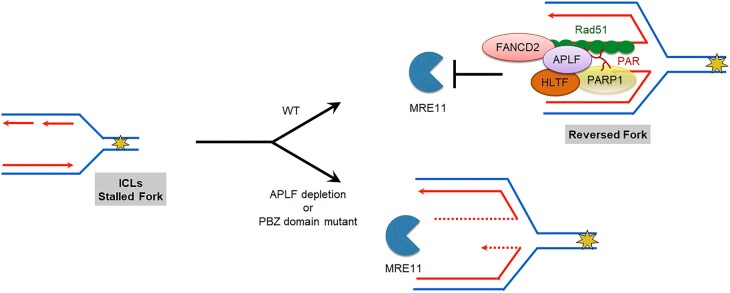

## Introduction

Aberrant DNA replication is a major source of mutations and genomic instability often associated with human diseases such as cancer (1). Faulty DNA replication can be caused by gene mutations in DNA replication machinery, aberrant chromosome structures such as hairpin or cruciform secondary structures, and DNA lesions arising from exogenous or endogenous insults. As a result, DNA replication forks stall often trigger DNA double-strand breaks (DSBs), recombination, and chromosome rearrangements, eventually resulting in genome instability. To prevent such situations, cells have evolved fork protection mechanisms, known as fork reversal, to minimize the genotoxic effects of replication stress by stabilizing, repairing, and restarting stalled forks, which represent important barriers to prevent tumorigenesis (2–4). Paradoxically, these mechanisms also act in cancer cells to contribute to chemoresistance against various DNA-damaging agents (5).

Fork stability prevents stalled forks from collapsing into poisonous DSBs (4). Currently, fork stability is achieved by DNA translocase-mediated fork reversal, which is protected by the BRCA1/BRCA2/RAD51 axis and FANCD2 of Fanconi anemia pathway to prevent the nascent DNA degradation by DNA nucleases such as MRE11 (6–8). The depletion of BRCA1, BRCA2, RAD51 or FANCD2 results in fork degradation mediated by the MRE11 nuclease. Recent advances have revealed that PARP1 is a sensor of replication stress that recruits DNA translocases, including HLTF, SHPRH, ZRANB3 and SMARCAL1, to convert stalled forks into reversed forks, and this process also depends on RAD51 recombinase (4,9,10).

PARP1 is an ADP-ribosyl transferase that catalyzes the formation of poly (ADP-ribose) (PAR) polymers onto itself and its target proteins (11). Recent advances have revealed that PARP1 alone is an incomplete enzyme, and it requires HPF1 to complete PARP1 activity (12,13). PARP1 binds to HPF1 through the C-terminal L1013/W1014 residues of PARP1 (12,14,15). The PARP1/HPF1 complex targets serine residues for ADP-ribosylation, which are the main residues to be modified in response to DNA damage, and PARP1 and histones are major targets for ADP-ribosylation (12,13,16). In addition, the PARP1/HPF1 mediated poly-ADP-ribosylation (PAR) is counteracted by PARG and ARH3 (17,18), leading to the high turnover of PAR.

PARP1 can be activated by single-strand breaks (SSBs), double-strand breaks (DSBs), and stalled replication forks (19–25). Therefore, PARP1 is involved in base-excision repair (BER), nucleotide excision repair (NER), DSB repair and maintenance of fork stability. Like the other posttranslational modifications, PAR can be recognized and bound by several specific domains, including the macrodomain, BRCT domain, and PAR-binding zinc finger (PBZ) domain (11). The BRCT domain of BARD1 can interact with PAR, which is important for the BARD1 recruitment to DNA damage sites (26,27). ALC1, which contains the macro domain, is recruited to DNA damage sites by binding to the PAR chain (28). APLF containing the PBZ domain is recruited to DNA damage sites through binding to the PAR chains (29,30).

Previous studies have shown that APLF functions as a scaffold protein that facilitates DSB repair through non-homologous end joining (NHEJ) and SSB repair through base/nucleotide excision repair (BER/NER). APLF contains four domains: a forkhead-associated domain (FHA), a Ku-binding motif (KBM), a tandem of PBZ domains, and an acidic domain (AD). The N-terminal FHA domain interacts with phosphorylated XRCC1 and XRCC4 (31–33), the KBM domain interacts with KU80 (34,35), the PBZ domains interact with PAR polymers (29,30), and the C-terminal AD domain interacts with histones, which may function as histone chaperones (36–38). APLF facilitates NHEJ by promoting the formation and stability of complexes between KU70/KU80 and XRCC4-LIG4 on the DNA ends, which are required for efficient ligation, and the C-terminal AD domain is critical for bridging (39,40). In addition, the depletion of APLF sensitizes cells to various DNA-damaging agents, including methyl methanesulfonate (MMS), ultraviolet (UV), H_2_O_2_, and cisplatin (32,41). MMS is a DNA alkylation agent that induces N7-methylguanine, N3-methyladenine and N3-methylguanine (42), UV induces cyclobutane thymine dimers and (6, 4) photoproducts (43), H_2_O_2_ induces base oxidation and SSBs (44), and cisplatin majorly forms intrastrand crosslinks between d (GpG) and d (ApG) and interstrand crosslinks (ICLs) with a lower frequency (45,46). MMS, UV and H_2_O_2_-induced DNA lesions are majorly repaired by the BER/NER repair pathways, whereas cisplatin-induced ICLs are majorly repaired by the Fanconi anemia (FA) pathway (47). APLF responds to various DNA lesions and is recruited to DNA damage sites dependent on the PARP1 activity. The PARP1 inhibitors abolished this recruitment (41). Interestingly, an in-vitro protein reconstitution assay also revealed that APLF is critical for PARP1 activity (31).

In this study, we identify the novel function of APLF involved in ICL repair and fork protection. We demonstrate that cisplatin induces the accumulation of APLF at DNA damage sites and stalled forks, and the process depends on PARP1 activity. APLF facilitates the recruitment of FANCD2 to stalled forks. The depletion of APLF reduces the levels of FANCD2 at stalled forks simultaneously, resulting in fork degradation by MRE11 nucleases. Interestingly, we also find that cisplatin-resistant cancer cells exhibit higher expression levels of APLF and HR-related genes than their parental cell lines. The depletion of APLF sensitizes cells to cisplatin and results in fork instability. Our results reveal the novel function of APLF involved in ICL repair and fork stability.

## Materials and methods

### Cell culture

The bladder cancer cell line T24 (ATCC® HTB4™) and human osteosarcoma U2OS (ATCC® HTB-96™) cell line were maintained in McCoy's 5A media supplemented with 10% fetal bovine serum, 1% glutamine and 1% penicillin/streptomycin in a 37°C incubator with 5% CO2. The nasopharyngeal carcinoma cell line HONE1, HONE6 and HONE15 were maintained in RPMI medium supplemented with 5% fetal bovine serum, 1% glutamine and 1% penicillin/streptomycin in a 37°C incubator with 5% CO2. The human embryonic kidney cell line HEK293T (ATCC® CRL-3216™) was maintained in DMEM medium supplemented with 10% fetal bovine serum, 1% glutamine and 1% penicillin/streptomycin in a 37°C incubator with 5% CO_2_.

### RNA interference

The shRNA-lentivirus was generated by the transfection of HEK293T cells with packing plasmids, pCMVΔR8.91, pMD.G, and pLKO_TRC005 vector containing control shLacZ, shAPLF-1, shAPLF-2 and shFANCD2 sequences. All the plasmids are obtained by the RNAi core, Academia Sinica, Taiwan. The shLacZ targeting sequence is CGC GAT CGT AAT CAC CCG AGT (TRCN0000231722); the shAPLF targeting sequences are CAT CCT GGT GAT AGT GAT TAT (TRCN0000144122) and GCC CTA TAA TGC TCT TCA TAT (TRCN0000167011); shFANCM sequence is GAC TTC ATG AAA CTC TAT AAT (TRCN0000236647) and the shFANCD2 sequence is ATC ATG CAG CTG ATC AGT ATT (TRCN0000417689). Cells were infected with these shRNA-lentiviruses and selected with 2 μg/ml puromycin for one week. The pLKO_TRC018 vector containing shFANCM or shFANCD2 for double knockdown is selected with 1 mg/ml hygromycin for one week. The depletion of each gene was verified both by qRT-PCR and western blotting.

### Plasmid construction

The pCMV6-APLF-tGFP plasmid was purchased from Origene (RG207936). The HA-tagged, FLAG-tagged APLF, PBZ mutant, and ΔAD mutant were generated using In-Fusion HD Cloning Kit (Takara Bio). To generate HA-tagged APLF, the wild-type human APLF gene (pCMV6-APLF-tGFP) was amplified using PCR with two primers, 5′ CAG ATT ACG CAA GCT TCA TGT CCG GGG GCT TCG A 3′ and 5′ TAT CAT GTC TGG ATC CTC ATT TTC TTT TCA TAA ACC TTT TT (APLF-WT primer) and cloned into pCEP4-HA using In-Fusion HD Cloning Kit (Takara Bio). The shRNA-resistant APLF (pCEP4-HA-APLF, PBZ mutant and ΔAD) was generated using two primers, 5′ TCC AGG AGA CTC AGA CTA TGG AGG TGT ACA AAT CGT GGG 3′ and 5′ TCT GAG TCT CCT GGA TGG CTA AAA TGT TGA AAA TGA ACA GGA 3′ (shAPLF resistant primers). The pCEP4-HA-PBZ mutant was generated using two primers, 5′ AGC TCC CTA TGG ACC ATC CGC TTA TAG GAA GAA TCC CCA GCA CAA GA 3′ and 5′ GGT CCA TAG GGA GCT TCA GGC CGG TCA TCA GTC TCA TCT TGG CCC A 3′. The pCEP4-HA-APLF (ΔAD) was generated using two primers, 5′ CAG TGT GAT AGG TTT TAG ATG AAG ATA ATG ATA ATG 3′ and 5′ AAA CCT ATC ACA CTG GAA GCG TAT TAT GTC 3′. All the pLX304-FLAG-APLF (WT, mut, ΔAD) were generated using two primers 5′ GTA CAA AAA AGC TAG CAT GGA CTA CAA AGA CCA TGA CGG TG 3′ and 5′ TTA ACG CGC CAC CGG TTC ATT TTC TTT TCA TAA ACC 3′ and the pCEP4-HA-APLF, PBZ mutant, and ΔAD plasmids as a template, and cloned into pLX304-FLAG vectors. To generate the GST-tagged APLF, the pGEX6p-APLF (WT, PBZ mutant, ΔAD) were generated using two primers 5′ GGG ATC CCC GGA ATT CTC CGG GGG CTT CGA GCT 3′ and 5′ GAT GCG GCC GCT CGA GTC ATT TTC TTT TCA TAA ACC 3′ and the pLX304-FLAG-APLF, PBZ mutant, and ΔAD plasmids as a template, respectively, and cloned into the pGEX6p vector using In-Fusion HD Cloning Kit (Takara Bio). The various GST-APLF constructs were generated using In-Fusion HD Cloning Kit (Takara Bio) with the pGEX6p-APLF as a template and the following primers. The GST-APLF (1–110) was generated using two primers 5′ AAT GCA ATG AAC CTT AAG AAA CAG TCA AGT GC 3′ and 5′ AAG GTT CAT TGC ATT TCC ACT TCA GAG GG 3′. The GST-APLF (1–200) was generated using two primers 5′ AAA CCT TTA AGT ACC AGC AAT CAG TGG AGG 3′ and 5′ GGT ACT TAA AGG TTT TGA TCA CTT AAA TGT TCT 3′. The GST-APLF (201–445) was generated using two primers 5′ CGG AAT TCT CAG TAC CAG CAA TCA GTG GAG 3′ and 5′ GTA CTG AGA ATT CCG GGG ATC CCA G 3′. The GST-APLF (446–511) was generated using two primers 5′ CGG AAT TCA GAA ATG TTT TAG ATG AAG ATA ATG 3′ and 5′ CAT TTC TGA ATT CCG GGG ATC CCA G 3′. All of these constructs were verified by DNA sequencing.

### Generation of PARP1 and APLF-expressing cell lines

The pLHCX-PARP1-res plasmid carrying the sgRNA-resistant wild-type PARP1 was packaged into retrovirus particles using the Retro-X^TM^ Universal Packaging System (Takara Bio) in the GP2-293 cell line. The retrovirus-containing medium was harvested at 48 and 72 h after transfection and filtered through a 0.22 μm PES membrane syringe filter to eliminate packaging cells. PARP1-KO T24 cells were infected with the retrovirus with the presence of Polybrene (8 μg/ml, Sigma-Aldrich). 1 mg/ml hygromycin was added after 48 h of infection to select infected cells. The expression of PARP1 was verified by western blotting.

The HA-tagged APLF, PBZ mutant and ΔAD stably expressed T24 cells were generated by transfection with pCEP4-HA-APLF, PBZ mutant, and ΔAD into T24 cells, respectively. These plasmids are shAPLF-resistant against shRNA-mediated gene depletion. After 48 h of transfection, cells were selected with 1 mg/ml hygromycin for one week to generate the HA-APLF stably expressing cells. The resulting cells were further infected with shAPLF-lentivirus to deplete the expression of endogenous APLF. These cells were used for the DNA fiber and SIRF assay.

The FLAG-tagged APLF, PBZ mutant and ΔAD stably expressed T24 cells were generated by infection of lentiviruses containing the FLAG-APLF, PBZ mutant and ΔAD genes. These lentiviruses were generated by the transfection of HEK293T cells with packing plasmids, pCMVΔR8.91, pMD.G and pLX304-FLAG-APLF (WT, mut, ΔAD).

### SIRF assay

3 × 10^4^ cells were seeded in an 8-well chamber slide (Millipore) and pulse-labeled with 10 μM EdU (Sigma-Aldrich) for 15 minutes followed with 100μM cisplatin treatment for 3 h. The cytoplasmic fractions of the cells were removed by extraction buffer (25 mM HEPES [Sigma-Aldrich], pH7.4, 50 mM NaCl [Sigma-Aldrich], 3 mM MgCl_2_ [Sigma-Aldrich],1 mM EDTA [Sigma-Aldrich], 0.3 M Sucrose [Sigma-Aldrich], 0.5% Triton X-100 [Sigma-Aldrich]). Then, the cells were fixed with 4% paraformaldehyde (Alfa Aesar) in PBS for 30 min at room temperature. The click reaction was performed with 2 mM copper sulfate (Sigma-Aldrich), 10 μM biotin-azide (Sigma-Aldrich), and 100 mM sodium ascorbate (Sigma-Aldrich) in PBS for 40 min and then quenched with PBS containing 10% FBS and 0.1% Triton X-100 for 30 min. A primary antibody against biotin was used to detect nascent synthesized DNA, and the other primary antibody was used to detect the target proteins. The slides were incubated with two oligonucleotide-conjugated secondary antibodies, anti-rabbit PLUS (Duolink, Sigma-Aldrich, DUO92002) and anti-mouse MINUS antibodies (Duolink, Sigma-Aldrich, DUO92001) for 60 min at 37°C. Ligases and DNA polymerases were then applied to the samples according to the manufacturer's protocol (Duolink, Sigma-Aldrich). Finally, nuclei were stained with DAPI for 15 min at room temperature and analyzed using a Nikon Eclipse 80i microscope equipped with a Plan Fluor 40×/0.75 DIC M/N2 objective. The resulting images were then analyzed using ImageJ.

### Isolation of proteins on nascent DNA (iPOND)

iPOND was performed as described (48). HEK293T cells were infected with lentivirus containing Flag-tagged APLF (WT), PBZ mutant (mut), or ΔAD. At least 1 × 10^7^ of HEK293T cells per sample were pulse-labeled with 10 μM EdU for 10–15 min. For the thymidine chase experiments, cells were further incubated with 10 μM thymidine (Sigma-Aldrich) for 30 min after EdU-labeling. 150 ${\mathrm{\mu M}}$ cisplatin and 4 mM HU (Sigma-Aldrich) were used to treat cells for 2 h after EdU-labeling. Cells were crosslinked with 1% formaldehyde (Sigma-Aldrich) for 20 min, followed by quenching with 0.125 M glycine (Fisher chemical). Cells were then washed three times with PBS, permeabilized with 0.25% Triton X-100 in PBS for 30 min at room temperature, and washed twice with PBS (once with 0.5% BSA in PBS and once with PBS). EdU was conjugated with a biotin using the click reaction. Cells were incubated in the click reaction buffer (20 μM biotin-azide, 100 mM sodium ascorbate and 2 mM CuSO_4_ in PBS) for 2 h at room temperature. After washed twice with PBS (once with 0.5% BSA in PBS and once with PBS), cells were resuspended in lysis buffer (50 mM Tris–HCl, pH 8 and 1% SDS) supplemented with protease inhibitors (MD Biol). Chromatin was sheared by sonication using Diagenode/Bioruptor Plus with high intensity for 10 cycles of 30 s on and 30 s off in ice cold water bath, followed by centrifugation at 16 000×g for 10 min. Supernatants were diluted with 1:1 PBS (vol/vol) containing protease inhibitors and incubated overnight with streptavidin agarose beads. Beads were washed once with low salt buffer (0.1% SDS, 1% Triton, 20 mM Tris pH 8, 2 mM EDTA, 150 mM NaCl), once with high salt buffer (0.1% SDS, 1% Triton, 20 mM Tris pH 8, 2 mM EDTA, 500 mM NaCl), once with buffer (0.25 M LiCl, 1% NP-40, 1% sodium deoxycholate, 1 mM EDTA, 10 mM Tris pH 8), and once with TE buffer. Captured proteins were eluted by boiling beads for 25 min at 95°C in 2× Laemmli buffer. The samples were loaded onto an SDS-PAGE gel for western blot analysis. The images were captured with the iBright imaging system (Invitrogen).

### Western blotting

Cells were lysed in 1xRIPA buffer (50 mM Tris–HCl, pH 7.4, 150 mM NaCl, 0.25% deoxycholic acid, 1% NP-40, 1 mM EDTA). The lysates were then sonicated by sonication using Diagenode/Bioruptor Plus with high intensity for 5 cycles of 30 s on and 30 s off in ice-cold water bath, followed by the addition of Laemmli sample buffer (4% SDS, 125 mM Tris (pH 6.8), 40% glycerol, 0.01% Bromophenol blue, 10% 2-mercaptoethanol) and the boiling of samples for 5 min at 100°C. The protein concentration was determined by the Bradford protein assay (Bio-Rad Protein Assay Dye Reagent Concentrate, cat no. #5000006). Samples were separated by a SDS-PAGE gel and transferred onto PVDF membranes. The PVDF membranes were blocked with 5% skim milk in TBST (20 mM Tris, pH 7.6, 150 mM NaCl, 0.1% Tween 20) for 1 h at room temperature. Subsequently, the membranes were incubated with primary antibodies at room temperature for 1 h, followed by incubation with the horseradish peroxidase (HRP)-conjugated secondary antibodies at room temperature for 1 h. The primary antibodies used in this study are listed in Supplementary Table S1.

### Co-immunoprecipitation

8 × 10^5^ cells were transfected with plasmids expressing Flag-tagged APLF (WT), APLF (mut), or APLF (ΔAD) followed by mock or cisplatin treatment. Cells were lysed in Tween lysis buffer (10% glycerol, 100 mM KCl, 5 mM MgCl_2_, 20 mM Tris–HCl pH 8.0, 0.2 mM EDTA, 0.1% Tween-20, and protease inhibitor cocktail [MD Biol]). The cell lysates were sonicated using Diagenode/Bioruptor Plus with high intensity for 10 cycles of 30 s on and 30 s off in an ice-cold water bath followed by centrifugation at 16 000×g for 10 min. The supernatant was transferred into a clear tube and was precleaned using protein G-sepharose. The target protein complex was pulldown by anti-Flag (Sigma-Aldrich, F1804) at 4°C for 2 h, followed by protein G-sepharose at 4°C for 1 h. The beads were washed with Tween lysis buffer for 3 times, and the immunoprecipitates were mixed with 4× Laemmli sample buffer and then boiled for 10 min. The beads were spun by 1800×g for 1 min at room temperature, and then the supernatants were loaded onto an SDS-PAGE gel for western blot analysis. The images were captured with the iBright imaging system (Invitrogen).

### Sister chromatid exchange (SCE)

2 × 10^6^ HONE1, HONE6, and HONE15 cells were seeded in a 100-mm culture plate and incubated with 9 μg/ml 5-bromodeoxyuridine (BrdU) (Sigma-Aldrich) for 46 h. 0.1 μg/ml colcemid (Thermo Fischer Scientific, 15212012) were added to arrest the cells in metaphase for 1hr, and the cells were harvested to make cytogenetic suspensions. Subsequently, the cells were incubated in 75 mM KCl at 37°C for 10 min, and fixed by methanol/acetic acid with a 3:1 ratio. The fixed cells were then dropped onto slides to spread the chromosomes. After being stained with Hoechst dye 33258, the slide was exposed to UV light for 10 min, followed by Giemsa staining. Images were acquired by a Nikon Eclipse 80i microscope equipped with a Nikon Plan Apo 60×/1.40 Oil DIC objective. For each cell line, 50 metaphases were analyzed to determine the number of sister chromatid exchanges.

### Quantitative polymerase chain reaction (qPCR)

RNA was extracted using an RNA Mini Kit (Zymo research), and cDNA was generated using an iScript cDNA Synthesis Kit (BIO-RAD). The resulting cDNA samples were analyzed using the real-time PCR analysis (ABI StepOne Plus Real-Time PCR Systems). The real-time PCR was performed in a 20 μl reaction with 0.4 μl of 10 μM forward primer, 0.4 μl of 10 μM reverse primer, 10 μl SYBR green supermix (KAPA Biosystems, KK4603), and 1 μl cDNA. The real-time PCR was started at 95°C for 3 min, followed by 40 cycles at 95°C for 3 s, and 60°C for 30 s. The relative mRNA expression levels of each gene were normalized to the levels of β-actin. The primer sequences are listed as follows: RAD51 forward: 5′ CAG TGA TGT CCT GGA TAA TGT AGC 3′. RAD51 reverse: 5′ TTA CCA CTG CTA CAC CAA ACT CAT 3′. BRCA1 forward: 5′ AGC AGA ATG GTC AAG TGA TGA ATA. BRCA1 reverse: 5′ ACT GCT GCT TAT AGG TTC AGC TTT 3′. UBC13 forward: 5′ CAA TGG CAG CCC CTA AAG TA 3′. UBC13 reverse: 5′ GTC TTC CAC TGC TCC GCT AC 3′. BARD1 forward: 5′ AAA TTT GAA TGG GTA AAA GCA TGT 3′. BARD1 reverse: 5′ TAA TAA GGT TGT CCT TTG GAT GGT 3′. PARP1 forward: 5′ CCA AGC CAG TTC AGG ACC TCA T 3′. PARP1 reverse: 5′ GGA TCT GCC TTT TGC TCA GCT TC 3′. ACTB forward: 5′ AAA ACA ACA ATG TGC AAT ACA AGT 3′. ACTB reverse: 5′ CTT AGT TGC GTT ACA CCC TTT CTT 3′.

### Cell cycle analysis by flow cytometry (BrdU and 7AAD staining)

The cell cycle analysis was performed using a FITC BrdU Flow Kit (BD biosciences, 559619). Briefly, 10^6^ cells were seeded into a 100-mm culture plate and incubated with 10 μM BrdU at 37°C for 45 min. Cells were fixed, permeabilized with BD Cytofix/ Cytoperm Buffer at room temperature for 15 min, and washed with BD Perm/Wash Buffer. Samples were then treated with DNase at 37°C for 1 h, followed by staining with FITC-conjugated anti-BrdU antibodies and 7-AAD at room temperature for 20 min. Samples were analyzed using BD FACSCalibur Flow Cytometer.

### DNA fiber analysis

5 × 10^5^ cells were cultured in a 60-mm culture dish and pulse-labeled with 25 μM CldU for 30 min and 250 μM IdU for 30 min, followed by treatment with 2 mM HU for 5 h. The cells were then harvested, and their nuclear fractions were isolated with buffer A (10 mM HEPES, pH 7.9, 10 mM KCl, 1.5 mM MgCl_2_, 0.34 M sucrose, 10% glycerol). 2 μl samples of nuclear fractions were then plated onto glass slides and lysed with spreading buffer (0.5% SDS, 50 mM EDTA, 200 mM Tris, pH 5.5) for 4 min. For each slide, the droplet run slowly down the length of the glass slide when tilted at 25–40° by gravity. The DNA fibers were fixed in a 3:1 methanol/acetic acid buffer for 10 min, denatured with 2N HCl at room temperature for 1 hour, and then blocked in PBS buffer (1% BSA, 0.1% Tween20 in PBS). The rat anti-BrdU primary antibody (1:200, Abcam, ab6326) against CldU and the mouse anti-BrdU primary antibody (1:200, BD Biosciences, 347580) against IdU were added onto the slides and incubated at 4°C overnight. The goat anti-rat AlexaFluor-594 secondary antibody (1:500, Thermo Fisher Scientific, A-110007) and the anti-mouse AlexaFluor-488 secondary antibody (1:500, Thermo Fisher Scientific, A-11001) were added to the slides at room temperature for 90 min. Images were acquired by a Nikon Eclipse 80i microscope equipped with a Nikon Plan Apo 100×/1.40 Oil DIC objective. At least 100 fibers of each sample were analyzed by using NIS Elements D4.20.00 software (Nikon), and graphs were plotted with GraphPad Prism software (Version 7.0).

### Immunofluorescence microscopy

4–5 × 10^4^ cells were cultured in a 4-well chamber slide and treated with 100 μM cisplatin for 3 h. Cells were fixed with 4% paraformaldehyde for 30 min and permeabilized with 2% Triton X-100 in PBS at room temperature for 10 min. The fixed cells were then incubated in blocking buffer (10% FBS, 0.1% Triton X100 in PBS) for 1 h at room temperature and further incubated with primary antibody at 4°C overnight. The anti-mouse AlexaFluor-594 secondary antibody (1:500, Thermo Fisher Scientific, A-11005) and the anti-rabbit AlexaFluor-488 secondary antibody (1:500, Thermo Fisher Scientific, A-11008) were added to the slides for 60 min at room temperature. DAPI was used to stain nuclei. Images were captured using Olympus FV1000 confocal microscope or Zeiss LSM780 Confocal Microscope System. At least 150 cells were counted to quantify the intensity of the γH2AX, APLF, and FANCD2 foci using FV10-ASW software (Olympus Life Science) or ZEN 3.6 (ZEISS ZEN microscopy software, blue edition).

### Colony formation assay

10^3^ cells were plated into a 6-well culture dish in duplicate and chronically treated with cisplatin and carboplatin at the indicated concentrations. After incubation for 7–10 days, colonies were formed and stained with 1% crystal violet (in 25% methanol). The colonies were then counted using the GeneTools software program (Syngene). The colony formation was determined by the number of colonies of each treatment divided by the number of cells of the untreated controls.

### Cytotoxicity assay

3 × 10^3^ cells were seeded in a 96-well plate in triplicate and treated with cisplatin for 96 h. The cell cytotoxicity was determined by the MTT [3- (4,5-dimethylthiazol-2yl)- 2,5-diphenyltetrazolium bromide, Sigma-Aldrich] assay at 37°C for 4 h. The samples were measured at a wavelength of 595 nm. The cytotoxicity was determined by the absorbance reads of each treatment normalized to the untreated controls.

### Determining the genomic platinum concentration

3 × 10^7^ cells were plated into a 150-mm culture dish. Cells were treated with or without 80 uM cisplatin for 3 h the next day. Cells were resuspended in DNA extraction buffer (10 mM Tris pH 8, 10 mM EDTA, 100 mM NaCl, and 1% SDS) and incubated with proteinase K for 16–18 h until the solution became clear. The sample was mixed vigorously with phenol/chloroform and followed by centrifugation at 14 000 rpm for 10 min at room temperature. The clean aqueous layer was collected and combined with isopropanol, followed by centrifugation at 14 000 rpm for 10 min at room temperature. The precipitate was washed with 70% EtOH and dried at 37°C for 10 min. Then, the precipitate was resuspended with TE and incubated with RNase at 37°C for 1 h. DNA was precipitated by using ammonium acetate and 100% EtOH and followed by centrifugation at 14000 rpm for 10 min at room temperature. Samples were washed with 70% EtOH and dried at 37°C for 10 min. Finally, genomic DNA was suspended in a TE buffer. Cisplatin-DNA adducts were detected by Agilent 7500C ICP-MS.

### Tumor xenograft animal model

All of the experimental protocols involving mice were approved by the Institutional Animal Care and Use Committee of NCKU (IACUC No. 111294). Supervised animal facilities by a board-certified veterinarian and ensured compliance with all ethical protocols. We also confirm that the study is reported by ARRIVE guidelines (https://arriveguidelines.org). NOD-SCID mice (6–8 weeks, male) were purchased from the National Laboratory Animal Center (Tainan, Taiwan). The mice were anesthetized by subcutaneous injection of Zoletil 100 (Virbac). Stable shRNA-expressing cells (10^7^ T24-shLacZ or shAPLF cells) were resuspended in a solution containing 50 μl PBS and 50 μl Basement Membrane Matrix (BD biosciences) and directly injected into the right site via BD Insulin Syringes (30G 3/10 cc, BD biosciences). The body weights of xenograft model mice were measured twice weekly until sacrifice. All procedures complied with the animal care standards set forth by the guidelines of the NCKU Laboratory Animal Center (Tainan, Taiwan).

### Mouse specimens

Tumor samples from mice were paraffin-embedded and cut into 5 μm thick tissue sections. Sections were deparaffinized, rehydrated, incubated with target antibody APLF (1:100, Gene Tex, GTX87979) and γH2AX (1:100, Cell Signaling Technology, 9718), and stained by VECTASTAIN Elite ABC HRP Kit (Vector Laboratories) and DAB Peroxidase (HRP) Substrate Kit (Vector Laboratories) according to the manufacturer's protocols. Images were captured using a light microscope (Olympus BX51).

### GST pulldown assay

The expression of GST-APLF fusion proteins was induced by 1 mM IPTG in the *Escherichia coli Rosetta* at 30°C for 2 h. Cells were resuspended in STE buffer (10 mM Tris, pH 8.0, 1 mM EDTA and 150 mM NaCl) with protease inhibitors and were lysed by sonication using Diagenode/Bioruptor Plus for 10 cycles of 30 s on/30 s off in an ice-cold water bath. The cell lysates were then mixed with Triton X100 to a final concentration of 1% of Triton X100, rotated at 4°C for 30 min, and finally centrifuged at 12 000 rpm at 4°C for 10 min. The resulting supernatants were mixed with glutathione (GSH) beads (GE Healthcare, GE17-5132-01) and rotated at 4°C overnight. The GST fusion proteins were purified by washing the beads with PBS for 3 times.

For the GST pull-down assay, the purified GST-APLF fusion proteins were incubated with total cellular lysates derived from HEK293T cells and rotated at 4°C for 3 h. The beads were washed with wash buffer (50 mM Tris, pH 7.5, 150 mM NaCl, 1 mM EDTA, 0.1% Triton X-100) for 3 times. The proteins associated in the complex were separated by an SDS-PAGE gel and detected with specific antibodies.

### Statistical analysis

GraphPad Prism version 7.0 (GraphPad Software) was used for performing statistical analysis. The significance of quantitative data was assessed by the Mann–Whitney test and Student's *t*-test. Differences between tumor-growth curves were determined by repeated measures of two-way ANOVA. For all figures, *P < 0.05; **P < 0.01; ***P < 0.001. All experiments were carried out with *n* ≥ 2 biological replicates.

## Results

### APLF is highly correlated with pan-ADP ribose modification at DNA damage sites upon cisplatin treatment

**Figure 1.**
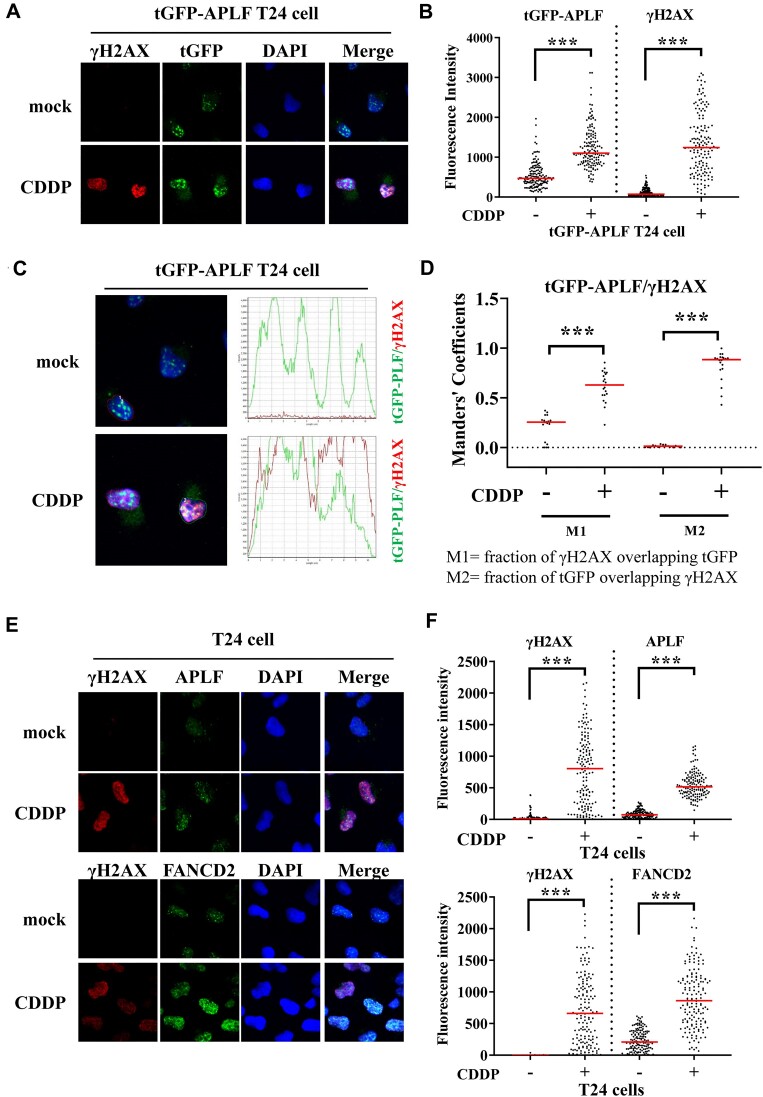
APLF and FANCD2 are enriched at DNA damage sites upon cisplatin treatment. (**A**) Representative images of immunofluorescent staining of tGFP-APLF (green) and γH2AX (red). Nuclei were stained with DAPI. T24 cells expressing tGFP-APLF were treated with mock or 100 μM cisplatin (CDDP) for 3 h. (**B**) Quantification of tGFP-APLF and γH2AX fluorescent intensity after cisplatin treatment. At least 150 cells from each sample were measured. The *p*-value was determined by the Mann–Whitney test. *** represents *P* < 0.001. (**C**) The tGFP-APLF and γH2AX intensities are highly correlated after cisplatin treatment. The profiles of tGFP-APLF and γH2AX fluorescent intensity in a cross-section are shown in the right panel. (**D**) The colocalization of γH2AX and tGFP-APLF was analyzed using Manders’ colocalization coefficient (M1 = red overlap with green; M2 = green overlap with red). At least 20 cells from each cell line were analyzed. (**E**) Representative images of immunofluorescent staining of APLF (green), γH2AX (red), and FANCD2 (green). (**F**) Quantification of APLF, γH2AX, and FANCD2 fluorescent intensity after cisplatin treatment. At least 150 cells from each sample were measured.

Recent advances have revealed that PARP1 functions as a sensor of replication stress and promotes the formation of reversed forks to prevent the collapse of forks into DSBs, in addition to its role in SSB repair (9,10,49–51). Since APLF interacts with PARP1, we investigate whether APLF is also involved in fork stability during replication stress. First, we tested whether APLF is localized at DNA damage sites. We transfected T24 cells with tGFP-tagged APLF and tested the localization of tGFP-APLF after cisplatin treatment using confocal microscopy. Cisplatin induces intrastrand and interstrand crosslinks between bases, blocking the progression of DNA replication, resulting in stalling of replication, and eventually inducing DSBs. As shown in Figure 1A and B, cisplatin treatment induced the accumulation of tGFP-APLF and γH2AX in the nucleus, and tGFP-APLF and γH2AX are highly colocalized, with approximately 90% of tGFP-APLF colocalized with γH2AX (Mander's coefficient M2) (Figure 1C, D), suggesting that APLF is enriched at DNA damage sites.

Previous studies have shown that the PBZ domain of APLF interacts with the ADP-ribose chain (29,30). To test the colocalization of PARP1, tGFP-APLF and ADP-ribosylation, we employed confocal microscopy with specific antibodies against PARP1, tGFP and ADP-ribosylation. The pan-ADP-ribose antibody was used to detect mono-, oligo, and poly-ADP-ribose (52). We found that ADP-ribosylation already occurred in the absence of cisplatin treatment and cisplatin treatment did not show significant differences in the levels of ADP-ribosylation over time (Supplementary Figure S1A, B). By quantitative colocalization analysis (Manders’ colocalization coefficient) of tGFP-APLF and pan-ADP-ribose, we found a significant increase of tGFP-APLF overlapped with pan-ADP-ribose following CDDP treatment over time, with approximately 60% of tGFP-APLF overlapped with pan-ADP-ribose at a 3-h time point following cisplatin treatment (Manders’ colocalization coefficient M2) (Supplementary Figure S1B).

Different from APLF and pan-APD-ribose staining, PARP1 was distributed throughout the nucleus and accumulated in several patch patterns which were enriched in nucleoli as previously described (Supplementary Figure S1C) (53). Cisplatin treatment induced the release of PARP1 from nucleoli to nucleoplasm and no clear foci formation was observed (Supplementary Figure S1C). Although it seems that we cannot determine the colocalization between PARP1 and APLF using confocal microscopy, the redistribution of PARP1 from nucleoli to nucleoplasm following cisplatin treatment also indicates that PARP1 localizes to DNA damage sites.

We next tested the localization of endogenous APLF following cisplatin treatment. Similarly, we found that APLF accumulated in the nucleus after cisplatin treatment (Figure 1E, F). APLF was highly colocalized with γH2AX and FANCD2 as revealed by the confocal microscopy, with approximately 50% of APLF and 80% of FANCD2 colocalized with γH2AX following cisplatin treatment (Manders’ colocalization coefficient M2) (Supplementary Figure S2A, B).

### APLF depletion impairs in repairing of cisplatin-induced DNA lesions

**Figure 2.**
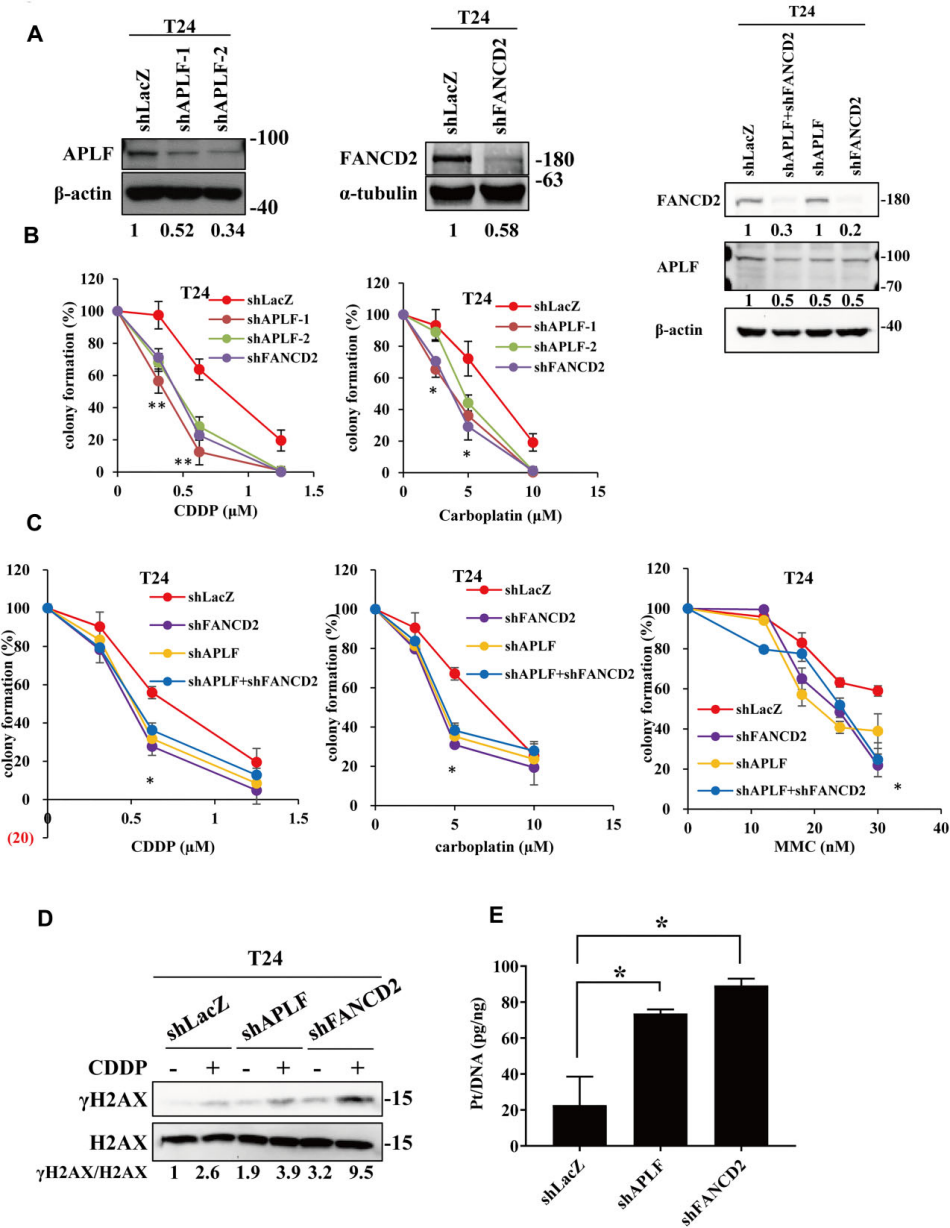
The depletion of APLF and FANCD2 impairs ICL repair. (**A**) The single depletion of APLF and FANCD2 and double-depletion both of APLF and FANCD2 were verified by western blot analysis with the specific antibodies as indicated. The expression of APLF and FANCD2 was depleted using shRNA-lentivirus. The non-targeting shLacZ was used as a control. (**B**, **C**) The colony formation assay. Cells were chronically treated with various concentrations of cisplatin, carboplatin, or MMC for 10 days. All data are the means ± standard deviation (SD) for at least two independent experiments. The *p-*value was determined by the Student's *t*-test. *P < 0.05; ** *P* < 0.01. (**D**) The immunostaining of γH2AX and H2AX of each cell line after cisplatin treatment. Cells were treated with mock or 40 μM cisplatin (CDDP) for 3 h. (**E**) Genomic platinum levels of each cell line were determined by ICP-MS. All data are the means ± SD for two independent experiments. The *P*-value was determined by the Student's *t*-test. *P < 0.05.

To test whether APLF is involved in repairing cisplatin-induced DNA lesions, we depleted the expression of APLF using shRNA-lentivirus. These shRNAs were able to deplete the expression of APLF significantly (Figure 2A). We found that the APLF-depleted T24 cells were more sensitive to cisplatin treatment than the control shLacZ cells (Figure 2B). The FANCD2-depleted cells were more sensitive to cisplatin than the APLF-depleted cells (Figure 2C and Supplementary Figure S3). The double depletion of APLF and FANCD2 did not further sensitize cells to these DNA-damaging agents compared to the FANCD2 single knockdown cells, suggesting that FANCD2 is epistatic to APLF (Figure 2C and Supplementary Figure S3). In addition to cisplatin treatment, these gene-depleted cells were also sensitive to carboplatin and mitomycin C (MMC) treatment, suggesting that APLF is involved in ICL repair (Figure 2B, C and Supplementary Figure S3).

Next, to test whether APLF facilitates ICL repair, we treated cells with 40 μM cisplatin for 3 h. The levels of DNA lesions were measured by γH2AX. We found that APLF or FANCD2 depletion increased γH2AX levels in cisplatin treatment, compared to the shLacZ control cells, indicating that APLF facilitates the repair of cisplatin-induced DNA lesions (Figure 2D). Since cisplatin is a platinum (Pt)-based drug, we further used Inductively Coupled Plasma Mass Spectrometry (ICP-MS) to determine the Pt levels in the APLF-depleted cells. The FANCD2-depleted cells were also included since FANCD2 is a crucial enzyme of the Fanconi anemia pathway to repair cisplatin-induced DNA lesions. Here, we examined the cisplatin accumulation in the shLacZ, shAPLF, and shFANCD2 T24 cell lines. The cells were treated with 40 μM cisplatin for 3 h, and the platinum levels of DNA extracted from cells were determined by ICP‐MS. The mean levels of platinum in shLacZ, shAPLF, and shFANCD2 were 22.7, 73.7 and 89.3 Pt (pg)/DNA (ng) (Figure 2E). Therefore, the depletion of APLF and FANCD2 showed significantly higher Pt levels in DNA than the control shLacZ cells, suggesting APLF facilitates ICL repair.

### APLF interacts with PARP1, HLTF, FANCD2 and RAD51

**Figure 3.**
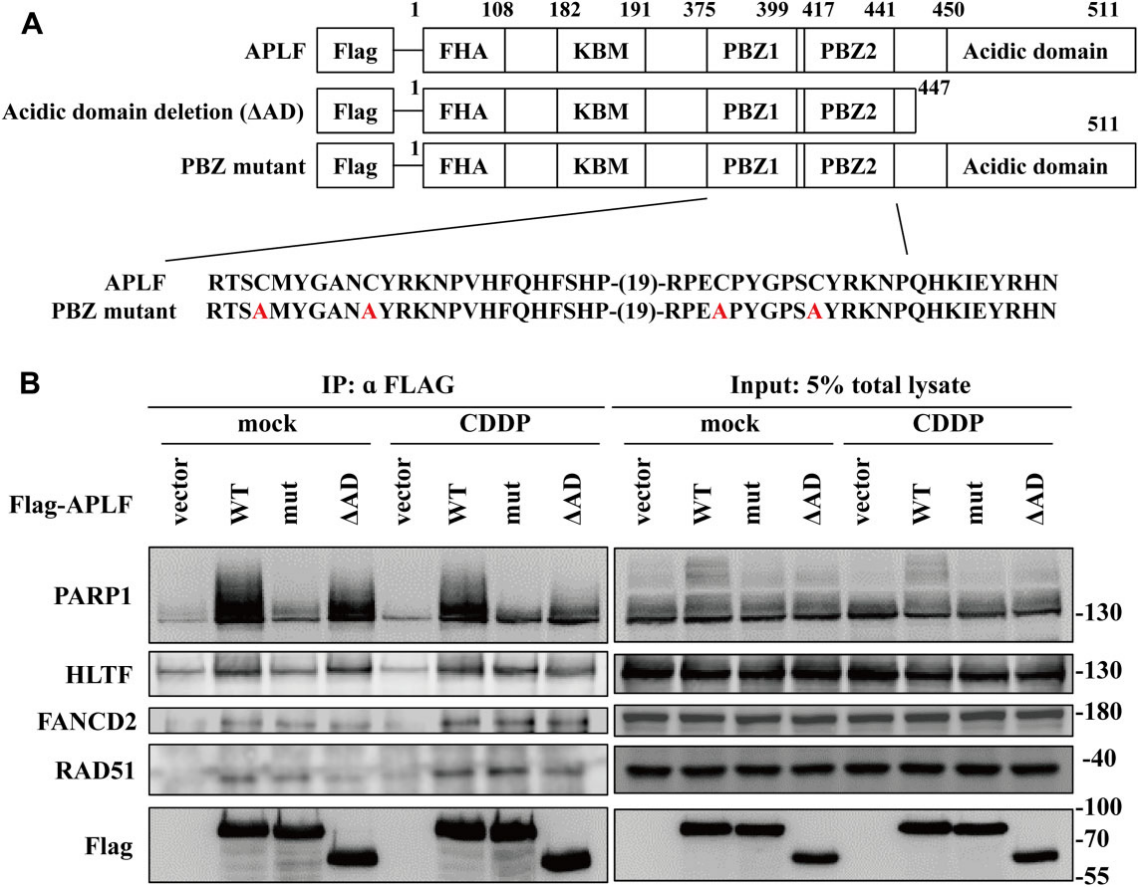
The PBZ mutant of APLF decreases the interaction with PARP1. (**A**) The schematic representation of APLF constructs. (**B**) The coimmunoprecipitation assay. HEK293T cells were transfected with various constructs of FLAG–APLF (WT, PBZ mutant, ΔAD), followed by mock or 100 μM cisplatin treatment for 2 h. The empty vector was used as a control. The FLAG-APLF complex was immunoprecipitated with an anti-FLAG antibody and the proteins were analyzed by western blotting. Input represents 5% of total cell lysates.

Previous studies have revealed that PARP1 interacts with APLF through the PAR moiety and the PBZ domains of APLF (29). Here, we generated the APLF mutant containing the C379A, C385A, C421A and C427A mutations in the tandem PBZ domains, referred to as the PBZ mutant (Figure 3A). We also generated the ΔAD mutant which is the deletion of the acidic domain of APLF (Figure 3A). HEK293T cells were transfected with plasmids containing the FLAG-tagged APLF, PBZ mutant, or ΔAD. To verify the interaction between APLF and PARP1, we performed a coimmunoprecipitation assay with an anti-FLAG antibody. APLF and ΔAD which contain the tandem PBZ domains interacted with PARP1 (Figure 3B). By contrast, the PBZ mutant significantly reduced the interaction with PARP1. Interestingly, cisplatin treatment did not further enhance the interaction between APLF and PARP1 (Figure 3B). We further found that APLF can interact with DNA translocase HLTF, FANCD2 and RAD51 in the presence or absence of cisplatin treatment, and the PBZ mutant and ΔAD did not disrupt these interactions (Figure 3B).

To verify our coimmunoprecipitation assay, we also purified the GST-tagged wild type, PBZ mutant, and ΔAD APLF fusion proteins from *Escherichia coli Rosetta* (Supplementary Figure S4A). We incubated the purified GST fusion proteins with HEK293T cell lysates. The proteins associated with GST fusion proteins were pulled down by glutathione sepharose beads. The GST protein alone was used as the control. Consistent with our coimmunoprecipitation assay, the wild-type APLF, and the ΔAD mutant interacted with PARP1, HLTF, FANCD2 and RAD51 in the absence or the presence of cisplatin treatment (Supplementary Figure S4A). The pulldown PARP1 showed a smearing pattern indicating that PARP1 is PARylated. In contrast, the PBZ mutant reduced its interaction with PARP1, HLTF, FANCD2 and RAD51. However, we cannot rule out the possibility that the reduced interactions were due to less amount of the GST-PBZ mutant fusion proteins (Supplementary Figure S4A).

To test whether these protein-protein interactions occur at endogenous levels, we performed the coimmunoprecipitation assay with an anti-APLF antibody. We found that APLF was able to pull down PARP1, HLTF, FANCD2 and RAD51 and cisplatin treatment did not affect these interactions (Supplementary Figure S4B).

We further mapped the interaction domains of APLF with HLTF. We generated the GST-tagged APLF fusion proteins with different domains. APLF (1–100) contains the FHA domain, APLF (1–200) contains the FHA and KBM domains, APLF (201–445) contains the two PBZ domains, and APLF (445–511) contains the acidic domain (Supplementary Figure S5A). These fusion proteins were purified from *Escherichia coli Rosetta*. We performed the GST pulldown assay as previously described. We found that APLF (201–445) containing the two PBZ domains majorly interacted with PARP1 (Supplementary Figure S5B, C). APLF (201–445) interacted with HLTF strongest, followed by the APLF (1–200), and APLF (1–100) failed to interact with HLTF (Supplementary Figure S5B, C). These results indicate that the PBZ domain majorly contributes to its interaction with HLTF, while the KBM domain also contributes to minor interaction. APLF (1–200), but not APLF (1–100) interacts with FANCD2, indicating that the KBM domain interacts with FANCD2 (Supplementary Figure S5B, C). APLF (1–100) and APLF (1–200) showed similar interaction strength with RAD51, indicating that the FHA domain interacted with RAD51 (Supplementary Figure S5B, C).

### PARP1 facilitates APLF recruitment to DNA damage sites

**Figure 4.**
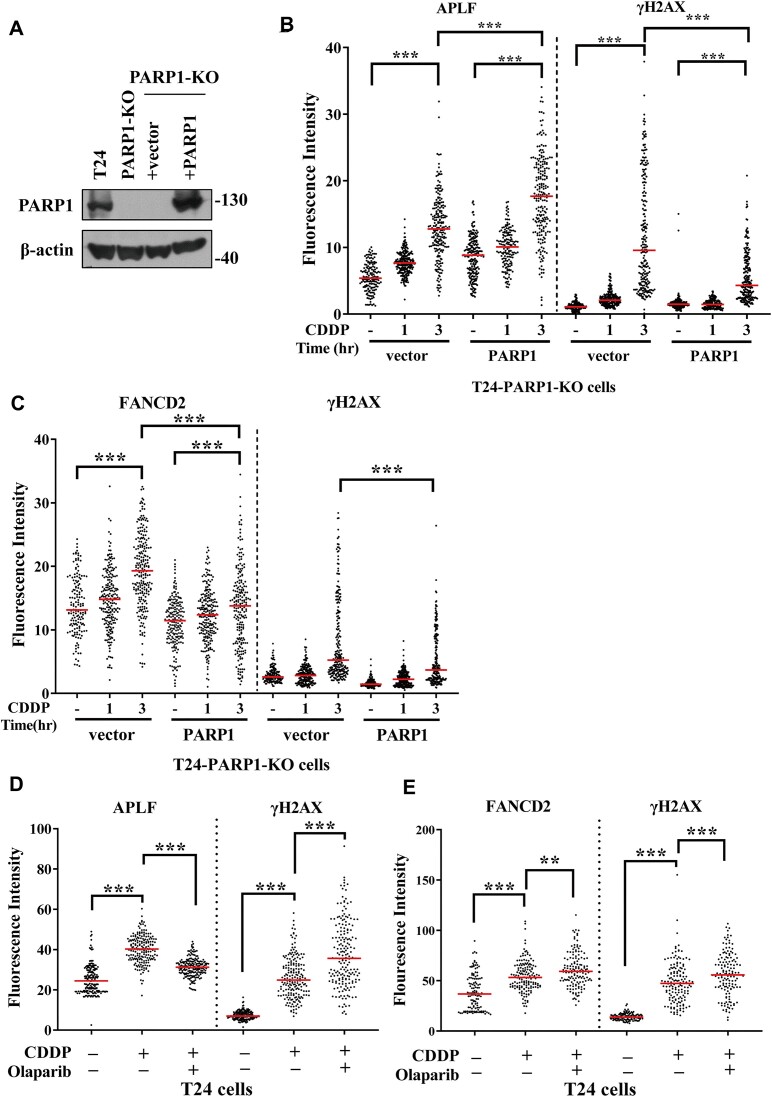
PARP1 activity facilitates APLF recruitment to DNA damage sites. (**A**) The immunostaining of PARP1 in the PARP1-proficient (T24) and knockout (PARP1-KO) T24 cells. PARP1 was also introduced into the PARP1-KO T24 cells using retroviruses. The pLHCX vector carrying sgRNA-resistant PARP1 was packaged into retrovirus particles in the GP2-293 cell line. The PARP1-KO cells were infected with the retrovirus to stably express wild-type PARP1. The empty vector was used as a control. (**B**) Quantification of APLF and γH2AX fluorescent intensity from the PARP1-deficient (vector) and PARP1-proficient cells following mock or 100 μM cisplatin treatment for 1 or 3 h. (**C**) Quantification of FANCD2 and γH2AX fluorescent intensity with a similar treatment to (C). (**D**) Quantification of APLF and γH2AX fluorescent intensity from T24 cells with mock or 100 μM cisplatin treatment for 1 h. 10 μM Olaparib was used to treat cells 2 h before cisplatin treatment. (**E**) Quantitation of FANCD2 and γH2AX fluorescent intensity with a similar treatment to (D). At least 120 cells from each condition were measured. The *P*-value was determined by the Mann–Whitney test. ** *P* < 0.01; ***P < 0.001; ns, not significant.

To test whether APLF recruitment to damaged sites depends on PARP1, we measured the APLF intensity in the nucleus in the PARP1-proficient and deficient cells using confocal microscopy. PARP1 knockout (PARP1-KO) T24 cells were generated using the CRISPR-based gene-knockout strategy (9). The PARP1-KO cells were then infected with retroviruses containing either empty vector (pLHCX) or wild-type PARP1 (pLHCX-PARP1) to generate the PARP1-deficient and PARP1-proficient cells, respectively (Figure 4A). These cells were then treated with cisplatin for 1 or 3 h. We found that APLF and γH2AX intensity in the nucleus increased over time following cisplatin treatment both in the PARP1-deficient and proficient cells (Figure 4B). Significantly, the PARP1-proficient cells further increased the APLF intensity in the nucleus, while significantly reduced the γH2AX and FANCD2 intensity in the nucleus (Figure 4B, C and Supplementary Figure S6A, B). Our results suggest that PARP1 recruits APLF to facilitate DNA repair, reducing the γH2AX and FANCD2 intensity in the nucleus.

Olaparib is an inhibitor of PARP1. Cisplatin treatment increased the APLF intensity in the nucleus (Figure 4D). However, Olaparib treatment significantly reduced the APLF intensity in the nucleus while further increasing the γH2AX and FANCD2 intensity in the nucleus (Figure 4D, E and Supplementary Figure S6C, D). Our results suggest that PARP1 activity is important to recruit APLF to damaged sites to facilitate DNA repair. The impairment of APLF recruitment induces more DNA lesions, where γH2AX and FANCD2 are accumulated at damage sites.

Since the depletion or inhibition of PARP1 decreased APLF abundance but increased FANCD2 accumulation to cisplatin-induced damage sites, it indicates that there are additional pathways to recruit FANCD2, possibly through FANCM. Several lines of evidence have shown that FANCM recognizes and binds to ICL sites to recruit FA core complex (47). To test this idea, we depleted FANCM using shFANCM lentivirus, and the FANCM depletion was verified by western blotting (Supplementary Figure S7A). We found that the FANCM depletion significantly reduced FANCD2 intensity following cisplatin treatment (Supplementary Figure S7B-C), while APLF depletion did not have much effect on FANCD2 intensity (Supplementary Figure S7D, E). The FANCM depletion in the APLF-depleted cells significantly reduced the FANCD2 intensity following cisplatin treatment (Supplementary Figure S7D, E), suggesting that FANCM is the major pathway to recruit FANCD2.

### APLF and FANCD2 accumulate at stalled replication forks

**Figure 5.**
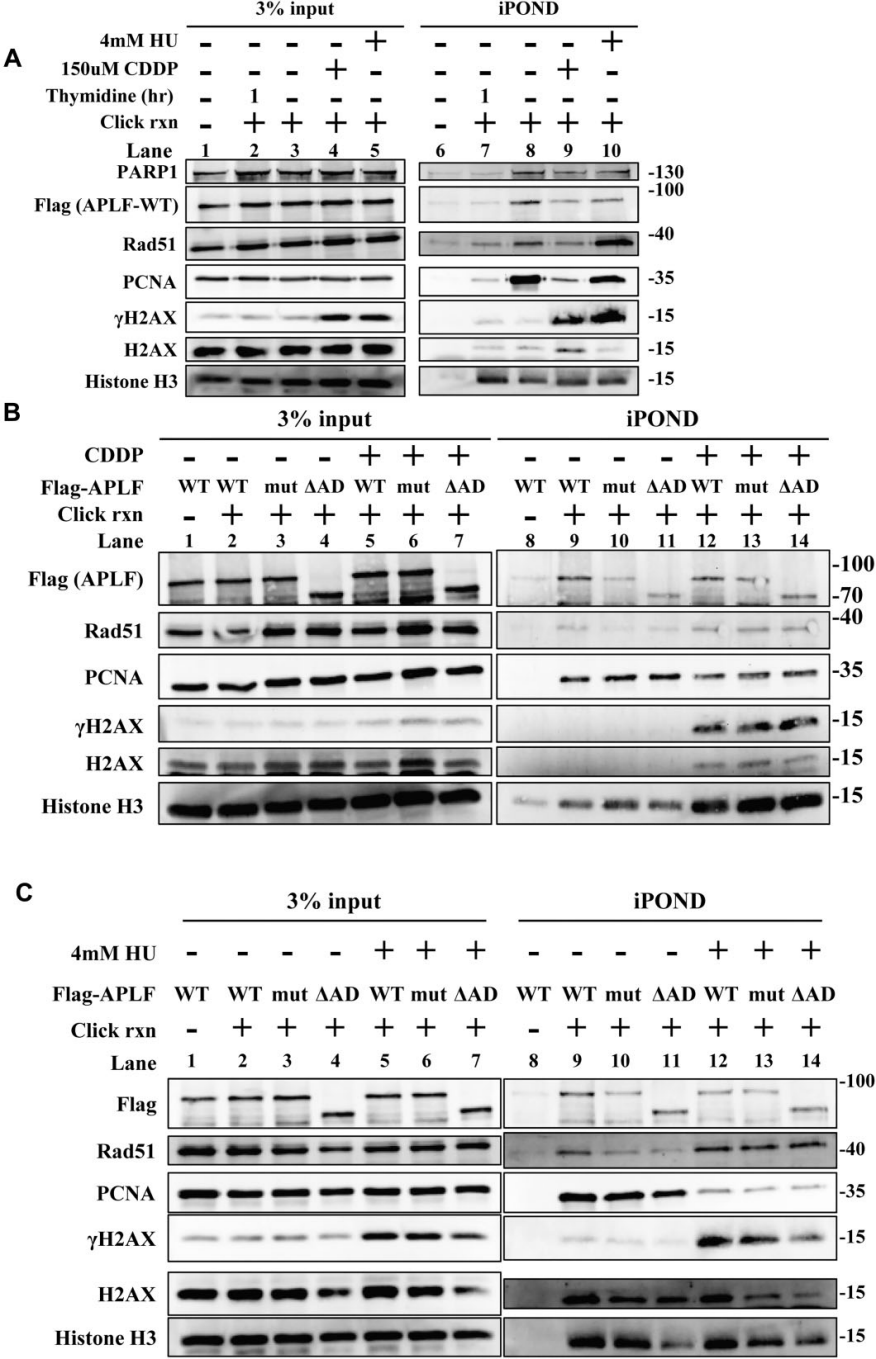
APLF is associated with replication forks. (**A**) HEK293T cells were infected with lentiviruses carrying FLAG-tagged APLF (WT). Cells were labeled with EdU for 10 min, followed by a thymidine chase for 1 h, 150 μM cisplatin treatment or 4 mM HU for 2 hr. The iPOND-captured proteins were analyzed by western blotting. The no-click control is processed without biotin-azide. Input represents 3% of total cell lysates. (**B**, **C**) HEK293T cells were transfected with plasmids carrying FLAG-APLF (WT), PBZ mutant (mut), or ΔAD, followed by mock, 150 μM cisplatin or 4 mM HU treatment for 2 h.

Since FA is the major pathway to repair cisplatin-induced ICLs during S-phase and PARP1 is associated with stalled forks, we wanted to test further whether APLF is associated with stalled forks. As APLF and FANCD2 did not reveal any foci formation similar to PCNA using confocal microscopy, it appears that the association of APLF or FANCD2 with replication forks cannot be easily revealed by confocal microscopy. To solve this problem, we performed the isolation of proteins on nascent DNA (iPOND) assay to test the association of APLF with replication forks (48). To ensure the success of the iPOND experiments, we transfected HEK293T cells with FLAG-tagged APLF. The HKE293T cells were pulsed-labeled with 10 μM EdU for 10 min, representing ongoing forks (Supplementary Figure S8A). Cisplatin and hydroxyurea (HU) were used to induce stalling of replication forks. We also include the negative control experiments in which cells were pulsed-labeled with 10 μM EdU for 10 min, followed by thymidine chase for 1 h. Under this condition, EdU tracks were away from replication forks (Supplementary Figure S8A). EdU was then conjugated with biotin using the click reaction. The proteins associated with replication tracks can be pulled down using the streptavidin-conjugated sepharose beads, and the associated proteins were analyzed by western blotting. Samples without the click reaction were used as the negative control. As shown in Figure 5A, PCNA is associated with ongoing replication forks in the EdU pulse-labeled samples (lane 8), but not in the no-click samples (lane 6). The thymidine phase for 1 hour significantly reduced PCNA levels (lane 7), suggesting EdU tracks were away from replication forks. Cisplatin and HU-induced stalled forks were verified by the enrichment of γH2AX in the cisplatin and HU-treated samples (lane 9–10) (Figure 5A). Cisplatin treatment for 2 h significantly reduced the PCNA levels (lane 9), indicating the collapse of forks. HU treatment for 2 h did not much affect PCNA levels, indicating that stalled forks are maintained (lane 10). These results were consistent with previous reports (54). These control experiments validated our iPOND experiments. Importantly, we found that FLAG-APLF was associated with ongoing replication forks (lane 8), as well as with cisplatin and HU-treated samples (lanes 9–10) (Figure 5A). A similar trend was also found for PARP1 and RAD51 (Figure 5A). Our results suggest that APLF, PARP1, and RAD51 were associated with ongoing and stalled replication forks. Since PARP1 is associated with stalled forks (55), the enrichment of PARP1, APLF, and RAD51 in the EdU-pulse labeled samples may correspond to stalled forks induced by spontaneous DNA lesions including ROS-induced DNA damage and aberrant chromosome structures (see SIRF results).

We further tested whether the PBZ domains of APLF are critical for the recruitment to the replication forks using the iPOND assay. The wild-type APLF was able to associate with replication forks in the presence or absence of cisplatin treatment (Figure 5B, lanes 9 and 12), whereas the PBZ mutant significantly reduced its association with forks (Figure 5B, lanes 10 and 13). The ΔAD containing the PBZ domains of APLF was able to bind to replication forks (Figure 5B, lanes 11 and 14). We also verified our results using 4 mM HU to stall replication forks. We observed similar results (Figure 5C). Our results suggest that the PBZ domains of APLF are critical for the APLF recruitment to stalled forks.

**Figure 6.**
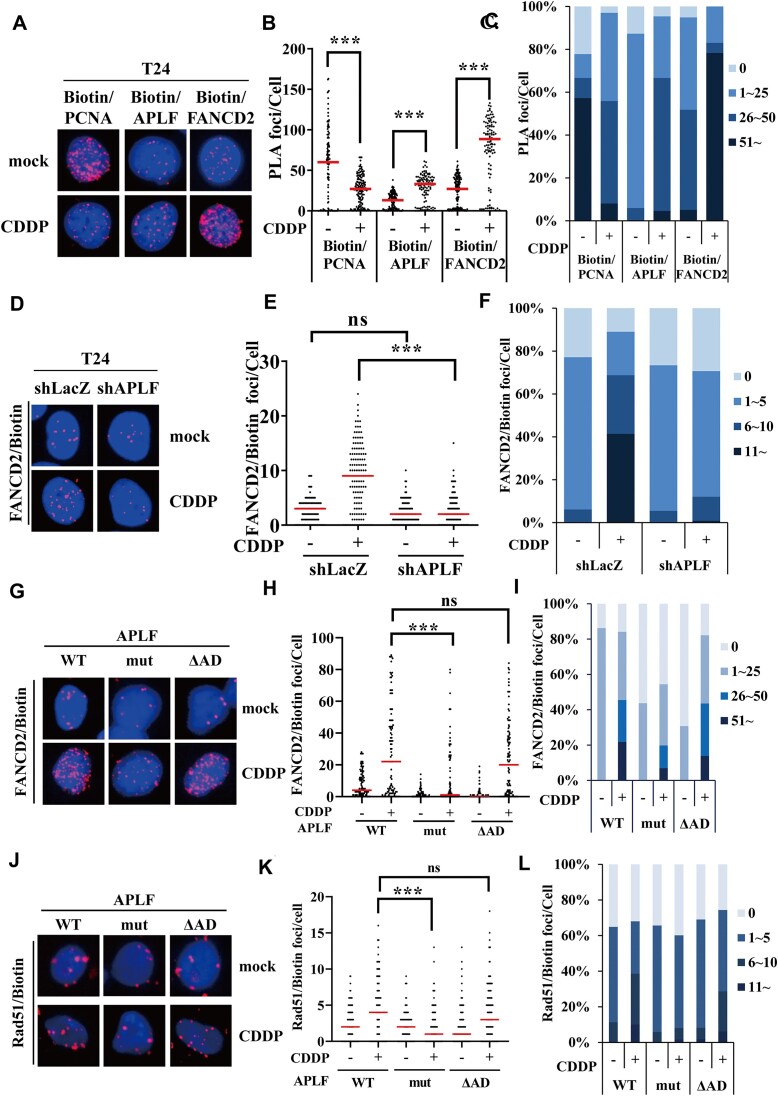
Cisplatin induces APLF and FANCD2 enrichment at stalled forks. (**A**) The representative images of PCNA, APLF, and FANCD2 SIRF assay. Cells were treated with mock or 100 μM cisplatin for 3 h. (**B**) Distribution of PLA foci from each condition derived from (A). (**C**) The percent stacked column graph is derived from (B). The number of PLA foci was classified into four groups: 0, 1–25, 26–50 and >51. (**D**) The representative images of FANCD2 SIRF assay in shLacZ and shAPLF T24 cells. Cells were treated similarly to (A). (**E**) Distribution of PLA foci from each cell line derived from (D). (**F**) The percent stacked column graph is derived from (E). (**G–J**) The representative images of FANCD2 and RAD51 SIRF assay. Cells were treated with mock or 100 μM cisplatin for 3 h. (**H–K**) Distribution of PLA foci from each condition. (**I–L**) The percent stacked column graph. The number of PLA foci was classified into four groups as indicated. At least 100 cells from each condition were measured. The *P*-value was determined by the Mann–Whitney test. ***P < 0.001; ns, not significant.

To verify iPOND results, we further employed the A Single-cell Assay for *in situ* Protein Interaction with Nascent DNA Replication Forks (SIRF) assay (56) (Supplementary Figure S8B). In this assay, T24 cells were labeled with EdU for 10 min and subsequently treated with mock or 100 μM cisplatin for 3 h to induce DNA damage. Next, biotin was conjugated to EdU using the click reaction. Specific antibodies against biotin and the target protein were used to detect the association of the target protein with replication tracks. Following the proximity ligation assay (PLA) protocols, if the target protein is associated with replication forks, it shows discrete foci in the nucleus, referred to as the PLA foci (56). Previously, we have validated our SIRF assay in which PCNA forms many discrete PLA foci in the nucleus, whereas EB1, a microtubule-associated protein, fails to show any PLA foci (9,57). Consistent with iPOND results, PCNA formed many discrete PLA foci in the nucleus in the absence of cisplatin treatment, with foci numbers ranging from 1 to >150 foci and approximately 80% of cells showed PCNA PLA foci, indicating that 80% of cells were in the S-phase (Figure 6A–C). Cisplatin treatment significantly reduced the PLA foci, which is consistent with the iPOND results (Figure 6A-C). At the same time, more cells (approximately 90% cells) showed PCNA PLA foci (Figure 6A–C), indicating that cisplatin treatment results in stalling DNA replication and accumulating cells in S-phase. These SIRF results suggest that the SIRF assay provides much more information than the iPOND assay.

APLF and FANCD2 already showed the PLA foci in the absence of cisplatin treatment, with approximately 50 PLA foci (Figure 6A–C), which is consistent with iPOND results. However, cisplatin treatment further increased APLF and FANCD2 PLA foci, with more than 50 and 100 PLA foci, respectively (Figure 6A–C), indicating that APLF and FANCD2 are enriched at stalled forks. We have to note that the iPOND is an ensemble method, in which the data come from hundreds of thousands of replication forks in millions of cells. It represents an average of proteins bound to replication forks but cannot distinguish the significant heterogeneity between each replication fork and cells (48). The SIRF assay measures the association of proteins with replication forks in a single-cell manner; therefore, the SIRF assay provides higher resolution than the iPOND assay. Since we observed that approximately 50 APLF and FANCD2 PLA foci have already formed in the absence of cisplatin treatment, we think that APLF and FANCD2 are associated with stalled forks, but not with every ongoing replication forks. It is based on the fact that PCNA formed more than 150 PLA foci in the absence of cisplatin treatment, which represents ongoing forks. By contrast, APLF and FANCD2 formed much fewer PLA foci than PCNA, with approximately 50 PLA foci at the same time. Therefore, our results suggest that APLF and FANCD2 are associated with stalled forks induced by spontaneous DNA damage arising from endogenous metabolites such as reactive oxygen species (ROS) or aberrant chromosomal structures. APLF and FANCD2 are enriched at stalled forks to resolve stalled forks.

### The PBZ mutant reduces FANCD2 and RAD51 recruitment to stalled forks

Since APLF accumulates at cisplatin-induced stalled forks, we further tested whether APLF facilitates the FANCD2 recruitment to the stalled forks. We depleted the expression of APLF using shRNA-lentivirus. The non-targeting shLacZ was used as a control. Cisplatin treatment significantly induced FANCD2 PLA foci, with >60% of cells showing >6 FANCD2 PLA foci in the nucleus (Figure 6D–F). By contrast, APLF depletion significantly reduced FANCD2 PLA foci, with only 10% of cells showing >6 PLA foci (Figure 6D–F). Similar results were also observed with different shAPLF sequence (Supplementary Figure S9A–C). In the U2OS cells, the APLF depletion also significantly reduced FANCD2 PLA foci in the nucleus (Supplementary Figure S9D–G).

To further test which domain of APLF contributes to FANCD2 recruitment, we infected T24 cells with retroviruses containing FLAG-APLF (WT), PBZ mutant and ΔAD to generate the APLF stably expressed cells and the association of FANCD2 with replication forks were determined by the SIRF assay. We found that APLF and ΔAD were able to facilitate FANCD2 recruitment to stalled forks after cisplatin treatment with a similar trend (Figure 6G–I). By contrast, the PBZ mutant significantly reduced the FANCD2 recruitment to stalled forks after cisplatin treatment (Figure 6G–I). A similar result was also found in the RAD51 SIRF assay (Figure 6J–L). Our results suggest that the PBZ domain is important to facilitate FANCD2 and RAD51 recruitment to stalled forks.

Since FANCM is essential to recruit FANCD2 to DNA damage sites, we also tested whether the FANCM depletion reduced the FANCD2 recruitment to stalled forks. Intriguingly, the FANCM depletion showed a similar FANCD2/biotin PLA foci number to the shLacZ control cells (Supplementary Figure S10). Our results suggest that the FANCD2 recruitment to DNA damage sites and stalled forks is through different mechanisms.

### PARP1 activity recruits APLF to stalled forks

**Figure 7.**
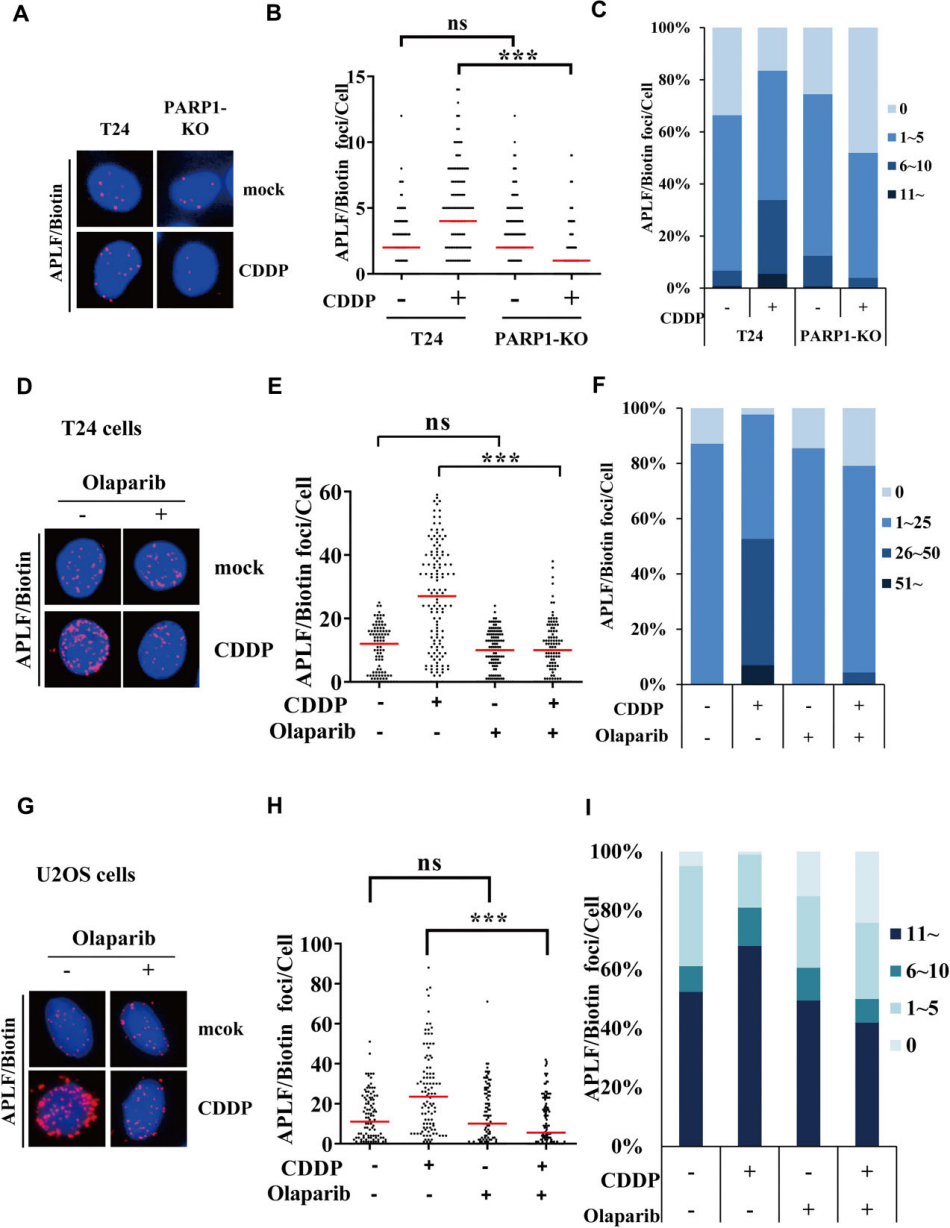
PARP1 recruits APLF to stalled forks. (**A**) The representative images of APLF SIRF assay in PARP1-KO T24 cells. Cells were treated with mock or 100 μM cisplatin for 3 h. (**B**) Distribution of PLA foci from each condition derived from (A). (**C**) The percent stacked column graph is derived from (B). (**D–G**) The representative images of APLF SIRF assay were derived from T24 and U2OS cells, respectively. Cells were pre-treated with 10 μM Olaparib for 2 h, followed by 100 μM cisplatin for 3 h. (**E–H**) Distribution of PLA foci from each condition. (**F–I**) The percent stacked column graph. At least 100 cells from each condition were measured. The *P*-value was determined by the Mann–Whitney test. ***P < 0.001; ns, not significant. All experiments have been repeated at least twice, with very similar results.

Since APLF contains the tandem PBZ domains that bind to PAR polymers, we tested whether APLF recruitment to stalled forks depends on PARP1 activity. We performed the APLF SIRF assay in the PARP1-KO cells. We found that the levels of APLF/biotin PLA foci were significantly reduced compared to the PARP1-proficient control cells (Figure 7A–C). Furthermore, we inhibited the PARP1 activity by pre-treatment cells with olaparib for 2 h, followed by cisplatin treatment for 3 h to induce DNA damage. Cisplatin treatment induced APLF PLA foci in the nucleus, with approximately 50% of cells showing >26 PLA foci (Figure 7D–F). By contrast, olaparib treatment significantly reduced the APLF PLA foci, with <5% of cells showing >26 PLA foci (Figure 7D–F). Similar results were also observed in U2OS cells (Figure 7G–I). Our results suggest that PARP1 activity is critical to recruit APLF to stalled forks.

### APLF facilitates fork protection through the PBZ domain

**Figure 8.**
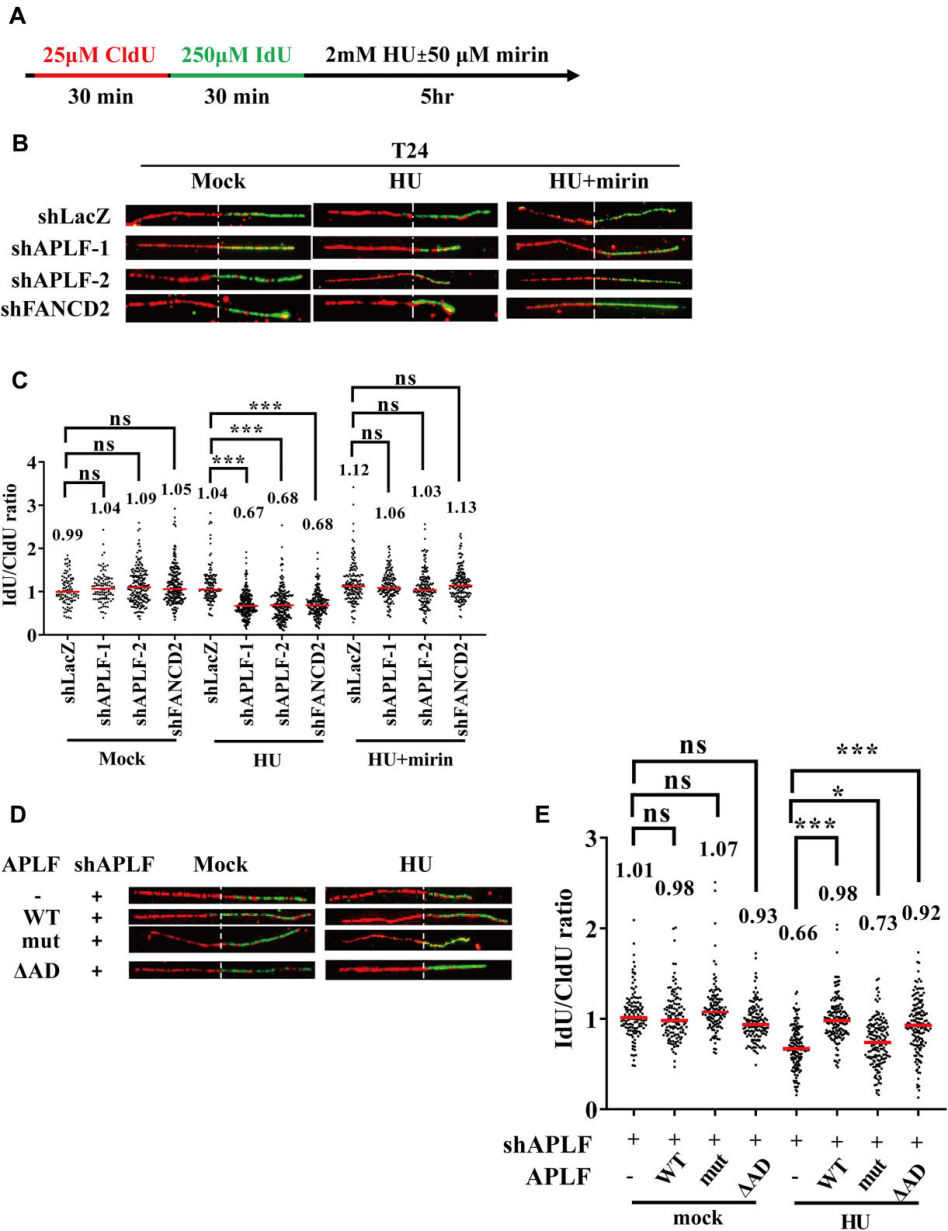
Depletion of APLF and FANCD2 significantly decreases IdU track length. (**A**) The labeling protocols for DNA fiber analysis. (**B**) Representative images of the DNA fiber analysis. (**C**) Quantification of track length ratios derived from each cell line. (**D**) Representative images of the DNA fiber analysis. APLF was transfected with plasmids carrying shRNA-resistant APLF (WT), PBZ mutant (mut), and ΔAD to generate the stably expressed cell lines, followed by the depletion of endogenous APLF using shRNA-lentivirus. (**E**) Quantification of IdU/CldU ratios derived from each cell line. At least 100 DNA fibers derived from each cell line were measured in these experiments. The *P*-value was determined by the Mann–Whitney test. *P < 0.05; **P < 0.01; ***P < 0.001; ns, not significant.

Several lines of evidence have shown that FANCD2 can facilitate replication fork stability by protecting the nascent DNA strands from degradation by the MRE11 nuclease (8). Since APLF facilitates FANCD2 recruitment to damaged forks, we want to test whether APLF has a similar function to protect nascent DNA from degradation. Therefore, we tested the fork stability using the DNA fiber assay. We labeled newly synthesized DNA with CldU for 30 min and IdU for 30 min, and then cells were treated with HU for 5 h to stall DNA replication (Figure 8A). The IdU track length was monitored for the stability of nascent DNA. As shown in Figure 8B-C, the depletion of APLF or FANCD2 did not affect the progression of replication in the absence of HU, suggesting that APLF and FANCD2 are not involved in DNA replication per se. In contrast to the absence of HU treatment, HU treatment resulted in further shortened IdU track length in the FANCD2-depleted cells compared to the shLacZ control cells. The inhibition of MRE11 activity by mirin restored the fork protection, suggesting that APLF and FANCD2 can protect nascent DNA from degradation by MRE11 (Figure 8B, C). Our results are consistent with previous publications, suggesting that FANCD2 protects stalled forks from degradation (8,58). We also found that the depletion of APLF resulted in further shortening of IdU track length, similar to the results derived from the FANCD2 depletion (Figure 8B, C).

To further verify the results derived from the DNA fiber assay, we used an alternative approach by labeling newly synthesized DNA with EdU for 15 min, followed by the treatment of cells with 2 mM HU for 5 h (Supplementary Figure S11). The EdU tracks were then conjugated with Cy5 using the click reaction, and the Cy5 fluorescence intensity can be monitored using confocal microscopy. The higher Cy5 fluorescence intensity represented longer EdU tracks and vice versa. Consistent with the DNA fiber assay, the APLF- and FANCD2-depleted cells also exhibited lower Cy5 fluorescence intensity than the control cells (Supplementary Figure S11). Therefore, we conclude that APLF protects nascent DNA from degradation by facilitating FANCD2 recruitment to stalled forks.

To further test which domains facilitate fork protection, we tested the PBZ mutant and the ΔAD mutant. By using DNA fiber assay, we found that the PBZ mutant, but not the ΔAD mutant, further reduced the IdU tracks compared to the wild-type APLF, suggesting that PBZ domains are critical to fork protection (Figure 8D, E).

### APLF depletion is sensitive to cisplatin treatment using a xenograft mouse model

**Figure 9.**
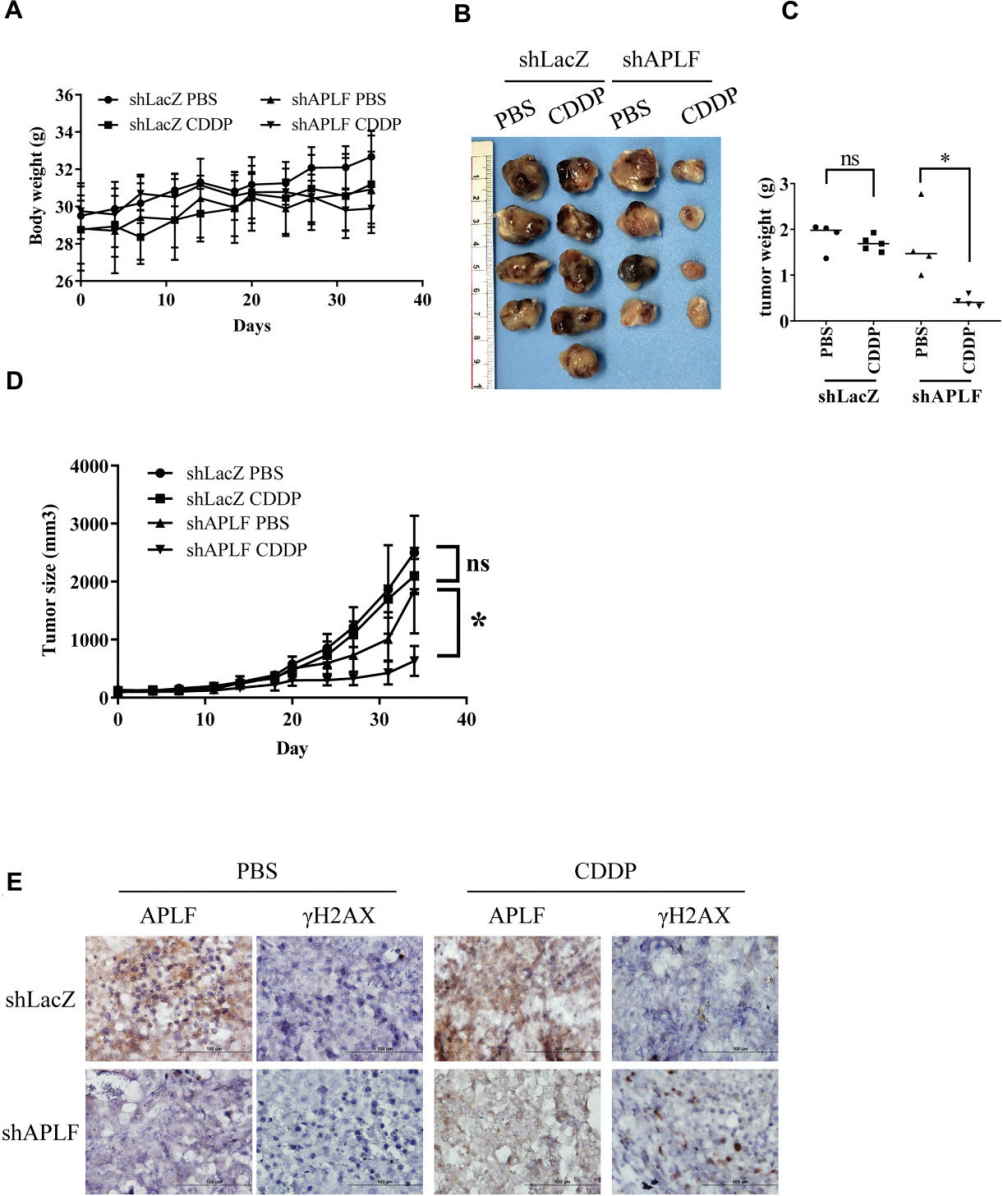
Depletion of APLF sensitizes cells to cisplatin in a xenograft mouse model. NOD SCID mice bearing shLacZ or shAPLF T24 xenograft tumors were treated with PBS or 2 mg/kg cisplatin for five weeks. (**A**) The body weight of each group during the 5-week treatment. (**B**) Tumor images representing excised tumors from each group. (**C**) Tumor weight for each group after sacrifice. (**D**) Tumor volume of each group during the 5-week treatment. The data are presented as means ± SD. * *P* < 0.05 represents a significant difference between the PBS and cisplatin groups. (**E**) Immunohistochemical staining of APLF and γH2AX in tumor tissues.

To test whether APLF depletion could inhibit tumor growth *in vivo*, we used a xenograft mouse model. The control and APLF-depleted T24 cells were mixed with Matrigel and injected subcutaneously into NOD-SCID mice. The mice were divided into four groups: (i) shLacZ PBS, (ii) shLacZ cisplatin, (iii) shAPLF PBS and (iv) shAPLF cisplatin. Mice were injected with PBS (nontreated control) or cisplatin (2 mg/kg) for 35 days, as described in the materials and methods section. The body weights and side effects among mice from PBS and cisplatin treatment did not show many differences, which indicated that treatment with cisplatin did not produce any apparent toxicity in mice (Figure 9A). However, the cisplatin treatment groups of shAPLF T24 cells showed the most significant growth inhibition effect (Figure 9B–D). In addition, we found that the APLF-depleted tumors showed higher γH2AX than the APLF-proficient groups following the cisplatin treatment (shLacZ cisplatin) (Figure 9E).

### Cisplatin-resistant nasopharyngeal carcinoma cells have high expression levels of APLF

Regaining fork protection has recently gained the spotlight as a potential marker for chemoresistance (59). Since APLF is important for fork stability, we wanted to test whether APLF is overexpressed in the cisplatin-resistant cell lines. The cisplatin-resistant nasopharyngeal carcinoma (NPC) cell lines, HONE6 and HONE15, were generated by chronic treatment of parental HONE1 cells with low-dose cisplatin (60,61). As shown in Supplementary Figure S12A, HONE6 and HONE15 cells are resistant to cisplatin treatment up to 20 μM of cisplatin, whereas HONE1 cells are sensitive to cisplatin, with 5μM cisplatin being sufficient to kill all HONE1 cells. Interestingly, we found that HONE6 and HONE15 cells showed elevated SCE compared to HONE1 cells (Supplementary Figure S12B, C) and several genes involved in HR pathways such as BRCA1, BARD1, BRCA2, RAD51 and FANCD2, were overexpressed compared to HONE1 (Supplementary Figure S12D-F). Since SCE is the result of chromosomal breaks and repaired by HR during S/G2-phase, it indicates that these cisplatin-resistant cells were able to overcome DSB-induced cell death and restore DSBs through HR. We found that APLF was overexpressed in HONE6 and HONE15 cells compared to HONE1 cells in both mRNA and protein levels (Supplementary Figure S12D, E). The depletion of APLF sensitized HONE6 cells to cisplatin and MMC (Supplementary Figure S13A–E). The APLF/FANCD2 double depletion had an additive impact on cellular sensitivity to cisplatin and MMC compared to FANCD2 single knockdown cells (Supplementary Figure S13C–E). Furthermore, the APLF depletion resulted in accumulation of cells in S-phase (Supplementary Figure S14) and the degradation of nascent DNA using the DNA fiber assay (Supplementary Figure S15). Similar results were also observed by the FANCD2 depletion. Our results suggest that elevated expression of APLF and HR-related genes in cisplatin-resistant HONE6 cells could contribute to cisplatin resistance through replication fork stability.

## Discussion

In this study, we reveal that APLF is involved in ICL repair as well as fork stability. First, we found that the APLF depletion sensitizes cells to cisplatin, carboplatin, and MMC (Figure 2B, C), exhibits high levels of γH2AX (Figure 2D), and impairs in removing Pt from DNA compared to APLF-proficient control cells (Figure 2E). Second, cisplatin induces the accumulation of APLF at DNA damage sites which is highly colocalized with pan-ADP-ribosylation (Figure 1A-F, Figure 4B, and Supplementary Figure S1A-B) and this process depends on PARP1 activity (Figure 4D). Third, we further identify that APLF is associated with stalled replication forks, as revealed by the iPOND and SIRF assay (Figure 5A–C and Figure 6B), and the association depends on PARP1 activity (Figure 7D-I). Fourth, the depletion of APLF significantly reduces FANCD2 association with stalled forks and increases the nascent DNA degradation by MRE11 nucleases (Figures 6D, E and 8A–E, and Supplementary Figure S11A–C), suggesting that APLF also plays an important role in fork stability. Recent advances have suggested that fork stability represents an important mechanism underlying chemoresistance (59). Consistent with this notion, we found that the depletion of APLF sensitizes cisplatin-resistant cancer cells to cisplatin and MMC, and increases nascent DNA degradation (Supplementary Figure S13 and Supplementary Figure S15A, B), suggesting that APLF confers ICL repair and fork stability to contribute to the cisplatin-resistant phenotypes of cancer cells.

Recent advances have revealed that serine residues are the major target for PARP1-mediated ADP-ribosylation during DNA damage (62–64), and PARP1 activity is regulated by HPF1 (12,14). The ADP-ribosylation already exists in the absence of DNA damage treatment and more than 150 proteins have been identified to be ADP-ribosylated, including PARP1, PARP2, histones, RFC1, FEN1, CHD1L, TOP2A, TOP2B, PRKDC, TOP1, RPA1, XRCC1 and HLTF, with PARP1 and histones being the major targets of ADP-ribosylation (13,65). Since these proteins are involved in DNA replication or replication stress, it suggests that ADP-ribosylation already occurs at replication forks. Consistent with this notion, we found that ADP-ribosylation occurs in the absence of DNA damage treatment (Supplementary Figure S1A, B and S16A). 1 mM H_2_O_2_ treatment induced pan-ADP-ribosylation (Supplementary Figure S16A). In contrast, 1.2 mM MMS or 100 μM cisplatin treatment induced lower levels of ADP-ribosylation (Supplementary Figure S16A), consistent with previous publications (66). Furthermore, 100 μM cisplatin treatment slightly reduced pan-ADP-ribosylation over time following cisplatin treatment, whereas γH2AX continuously increased (Supplementary Figure S16A, B). Our coimmunoprecipitation also indicates that less PARylated PARP1 was immunoprecipitated by APLF in samples treated with cisplatin for 2–3 h (Figure 3B and Supplementary Figure S4A). The dynamics of ADP-ribosylation following cisplatin treatment reflect the dynamic and complex regulation by PARP1/HPF1, PARG and ARH3 (17,18).

We identified several proteins to interact with APLF. These protein-protein interactions are verified by coimmunoprecipitation (Figure 3A-B), GST pull-down (Supplementary Figure S4A), and at the endogenous protein levels (Supplementary Figure S4B). First, consistent with previous results (29,30), the PBZ domain interacts with PARylated PARP1. We also found that APLF is highly correlated with pan-ADP-ribose foci following cisplatin treatment (Supplementary Figure S1A, B). Since HLTF, histones, and several proteins involved in replication stress are also ADP-ribosylated, it indicates that APLF can be recruited to stalled forks by interacting with these ADP-ribosylated proteins. Second, the PBZ domain of APLF contributes to the major interaction with HLTF, while the KBM domain contributes to the minor interaction with HLTF (Supplementary Figure S5A–C). Third, the KBM domain interacts with FANCD2 (Supplementary Figure S5A–C). Finally, the FHA domain interacted with RAD51 (Supplementary Figure S5A–C).

Interestingly, the PBZ mutant of APLF does not reduce its interaction with FANCD2 (Figure 3B). However, the FANCD2 recruitment to stalled forks is significantly reduced by the PBZ mutant according to the SIRF assay (Figure 6G–I). These results were not contradictory since the coimmunoprecipitation assay determines the protein-protein interactions, whereas the SIRF assay determines the association of a protein with replication forks. It indicates that APLF recruitment to stalled forks also depends on multivalent interactions, such as the interaction with ADP-ribosylated proteins through the PBZ domain. Since PARP1, BRCA1, BRCA2, RAD51, RPA are enriched at stalled forks (67) and FANCD2 forms complexes with PARP1, BRCA1, BRCA2, RPA, H2AX and RAD51 (68,69), APLF could function as a scaffold protein that mediates interactions with PARP1/FANCD2/RAD51/RPA/H2AX complexes. These multivalent interactions forming a liquid-liquid phase separation facilitate the FANCD2 recruitment to stalled forks. The liquid-liquid phase separation phenomenon commonly occurs in protein recruitment and localization in cells (70). Consistent with this idea, the structural analysis revealed that APLF is predominantly an intrinsically disordered protein and functions as a scaffold protein that mediates interactions with KU/DNA-PKcs, KU/XLF and KU/XRCC4/LIG4 complexes on DNA ends and establishes stable DNA synapses to facilitate NHEJ (34,35,39).

The APLF depletion resulted in the reduction of FANCD2 recruitment to stalled forks (Figure 6D-F), simultaneously with nascent DNA degradation (Figure 8A–C). The PBZ mutant of APLF significantly reduced FANCD2/biotin PLA foci (Figure 6G–I) and also resulted in nascent DNA degradation (Figure 8D, E). Two specific shRNAs result in similar results. Based on this evidence, we believe that the APLF function in stalled forks is specific, not due to off-target effects. Intriguingly, recent studies revealed that DNA-PKcs are recruited to active replication forks and promote fork reversal and slowing in response to replication stress (71), also implicating the specific function of APLF in stalled forks. The detailed mechanism of the DNA-PKcs-APLF interaction at stalled forks awaits further investigation in the future.

Although we found that the FANCD2 recruitment to stalled forks depends on APLF, the depletion or inhibition of PARP1 decreased APLF recruitment to DNA damage sites, while continuing to increase FANCD2 recruitment to damage sites over time according to confocal microscopy analysis (Figure 4C, E). It indicates that there are additional pathways to recruit FANCD2 to DNA damage sites, possibly through FANCM. Several lines of evidence have shown that FANCM recognizes and binds to ICL sites to recruit FA core complex (47). Indeed, we found that the FANCM depletion significantly reduced FANCD2 intensity following cisplatin treatment (Supplementary Figure S7B, C), while APLF depletion did not have much effect on FANCD2 intensity (Supplementary Figure S7D, E). The FANCM depletion in the APLF-depleted cells significantly reduced the FANCD2 intensity following cisplatin treatment, suggesting that FANCM is the major pathway to recruit FANCD2 to DNA damage sites. We also tested the effect of the FANCM depletion on FANCD2 recruitment to stalled forks. The FANCM depletion showed a similar FANCD2/biotin PLA foci number to the shLacZ control cells (Supplementary Figure S10A–C). Although FANCM is also found to be associated with stalled forks (67), our results indicate that the FANCD2 recruitment to stalled forks can be mediated through the other pathways, such as interactions with APLF and RAD51. APLF and RAD51 are highly enriched at stalled forks. Our results suggest that the FANCD2 recruitment to DNA damage sites and stalled forks is through different mechanisms.

Regaining fork protection has recently gained the spotlight as a potential marker for chemoresistance and this process depends on the stabilization of the RAD51-DNA filament (59). We found that the cisplatin-resistant nasopharyngeal carcinoma (NPC) cell lines, HONE6 and HONE15, show an elevated expression of APLF, PARP1, HR-related genes and FANCD2 when compared with its cisplatin-sensitive parental HONE1 cells (61). Consistently, these cisplatin-resistant cells show an elevated SCE, suggesting that cisplatin-induced DSBs are able to be repaired through HR efficiently. The depletion of APLF sensitizes these resistant cells to cisplatin, simultaneously with reduced FANCD2 and RAD51 at stalled forks and the degradation of nascent DNA. Our results are consistent with the notion that fork protection contributes to the chemoresistant phenotype. Therefore, targeting APLF could be a good therapeutic strategy to treat chemoresistant cancers.

### Limitations of the study

Our SIRF data was presented as foci numbers of each cell but not the fluorescence intensity of each cell. In addition, due to technical limitations, it is impossible to count EdU-SIRF and FANCD2-SIRF simultaneously in the same cells and normalize the FANCD2-SIRF data by the EdU-SIRF data of each cell. To overcome the technical limitations, we simultaneously performed the SIRF assay with the shLacZ control and shAPLF cells and observed a similar trend between each repeat. Although we cannot rule out the possibility that differences observed in the protein-EdU signal could also be caused by differences in EdU incorporation, we observed that the progression of DNA replication between shLacZ and shAPLF cells was similar in the absence of DNA damaging treatment according to the DNA fiber assay (Figure 8A–C). The distribution of FANCD2/biotin PLA foci between shLacZ and shAPLF cells was similar in the absence of cisplatin treatment (Figure 6D–F). Olaparib treatment alone did not affect the distribution of APLF/biotin PLA foci (Figure 7D–I). These data suggest that the APLF depletion or olaparib treatment did not affect DNA replication per se.

## Supplementary Material

gkae211_Supplemental_File

## Data Availability

All data generated or analyzed in this study are included in the article and its Supplementary Information.
