# Supplementary material for: APLF facilitates interstrand DNA crosslink repair and replication fork protection to confer cisplatin resistance
Source: Nucleic Acids Res. 2024 Mar 23;52(10):5676–97. doi: 10.1093/nar/gkae211 (PMC11162786; doi:10.1093/nar/gkae211)
Supplement: gkae211_Supplemental_File [file gkae211_supplemental_file.pdf]

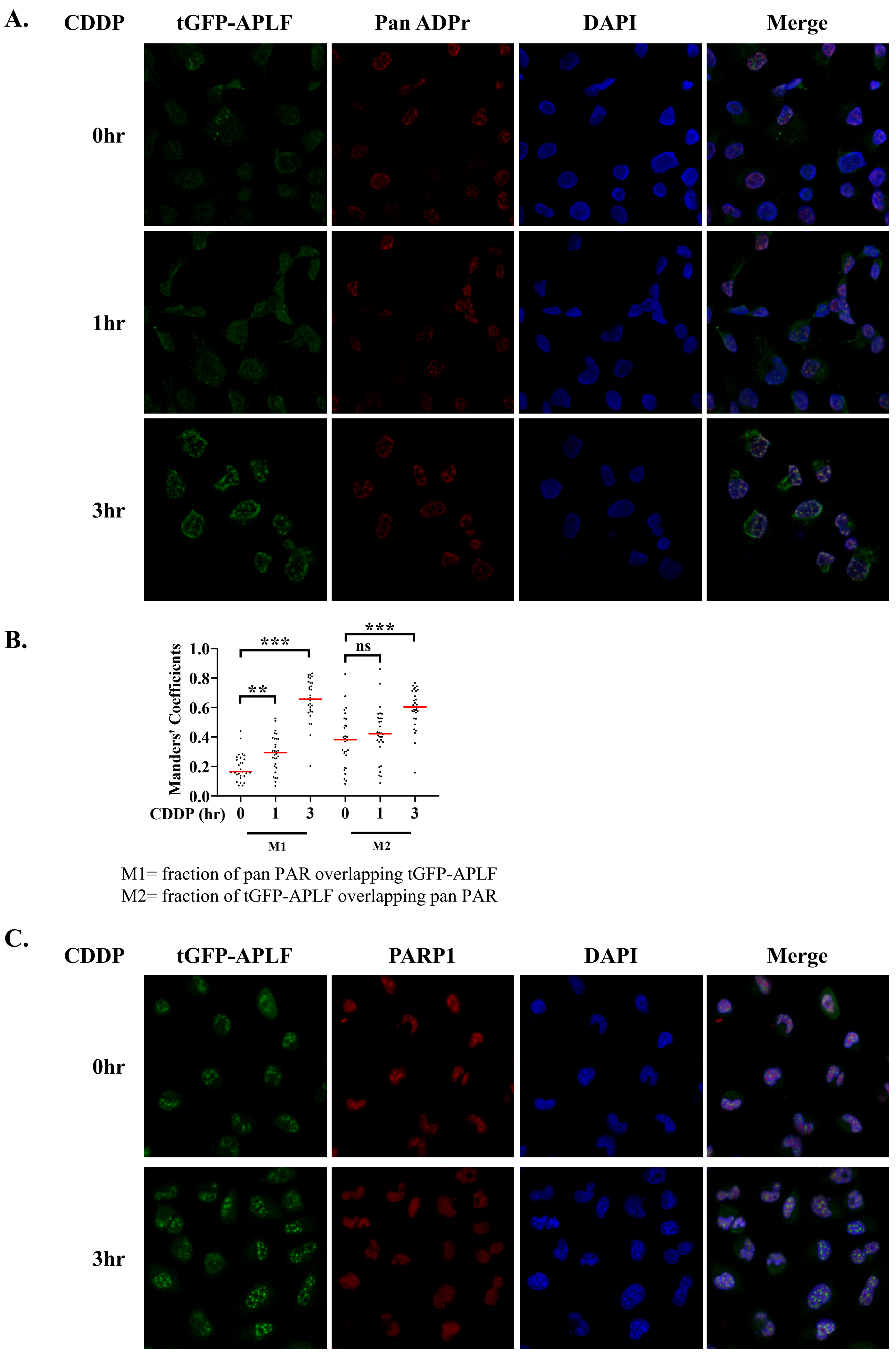
A.
CDDP
tGFP-APLF
Pan ADPr
DAPI
Merge
0hr
1hr
3hr
B.
Manders' Coefficients
1.0
0.8
0.6
0.4
0.2
0.0
***
**
***
ns
CDDP (hr)
0
1
3
0
1
3
M1
M2
M1= fraction of pan PAR overlapping tGFP-APLF
M2= fraction of tGFP-APLF overlapping pan PAR
C.
CDDP
tGFP-APLF
PARP1
DAPI
Merge
0hr
3hr

Figure S1. tGFP-APLF is highly colocalized with pan-ADP-ribose in the nucleus. (A) Representative images of immunofluorescent staining of tGFP-APLF (green) and pan-ADP-ribose (red). Nuclei were stained with DAPI. T24 cells expressing tGFP-APLF were treated with mock or 100 μM cisplatin (CDDP) for 1 or 3 hours. (B) The colocalization of tGFP-APLF and pan-ADP-ribose was analyzed using Manders' correlation coefficient (M1 = red overlap with green; M2 = green overlap with red). At least 30 cells from each sample were analyzed. The *p-value* was determined by the Mann–Whitney test. *** represents $p < 0.001$; ** $p < 0.01$; ns, not significant. (C) The representative images of tGFP-APLF and PARP1 in T24 cells expressing tGFP-APLF. Cells were treated with mock or 100 μM cisplatin for 3 hr.

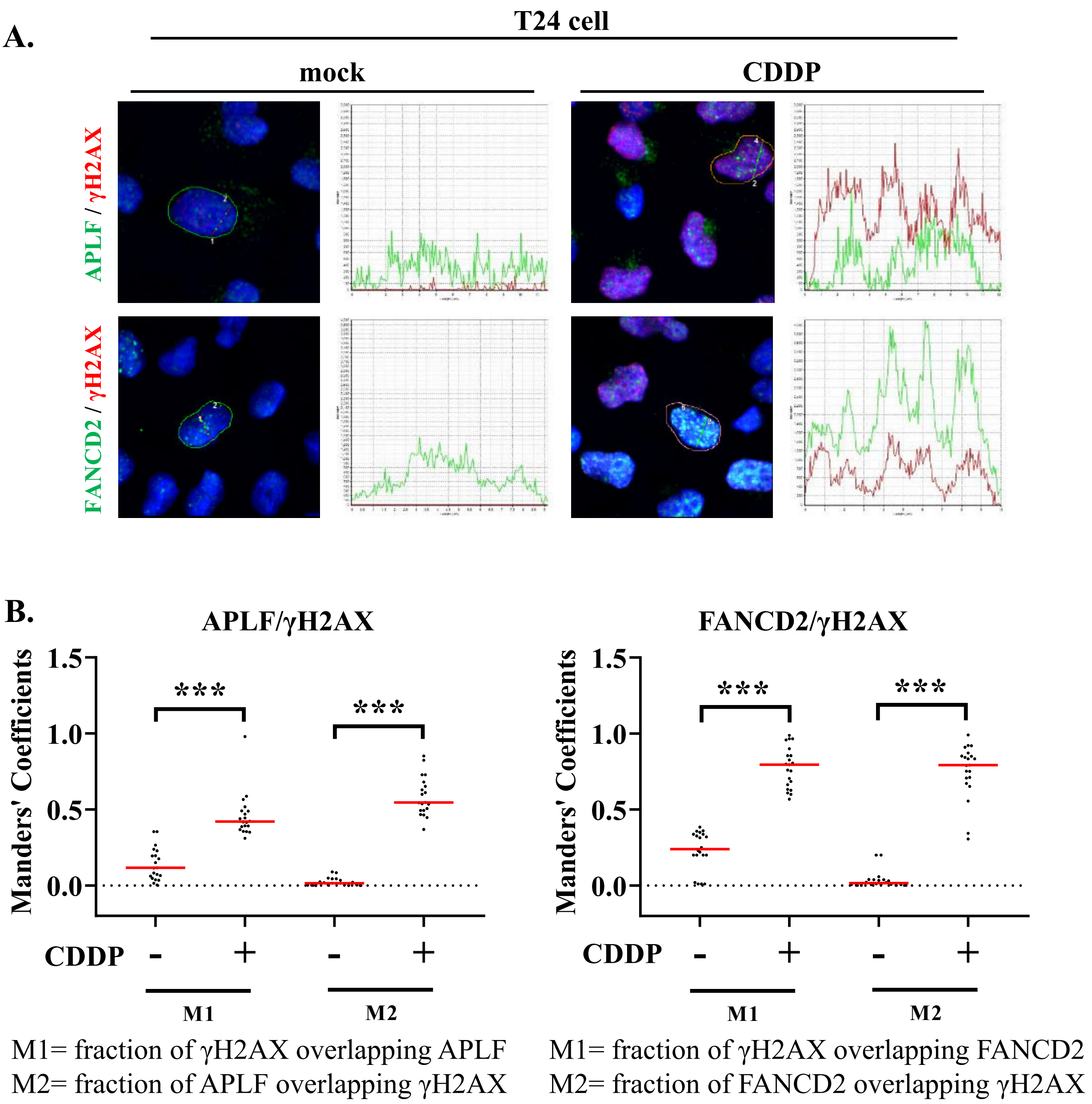

Figure S2. APLF, FANCD2, and γH2AX are highly correlated after cisplatin treatment. (A) Representative images of immunofluorescent staining of APLF, FANCD2, and γH2AX (left panel). The profiles of fluorescent intensity of each protein in a cross-section are shown in the right panel. Cells were treated with mock or 100 μM cisplatin for 3 hr. Cells were fixed and immunostained with anti-APLF, FANCD2, and γH2AX antibodies. (B) The APLF/ γH2AX and FANCD2/ γH2AX colocalization were analyzed using Manders' correlation coefficients (M1 = red overlap with green; M2 = green overlap with red). At least 20 cells from each sample were analyzed.

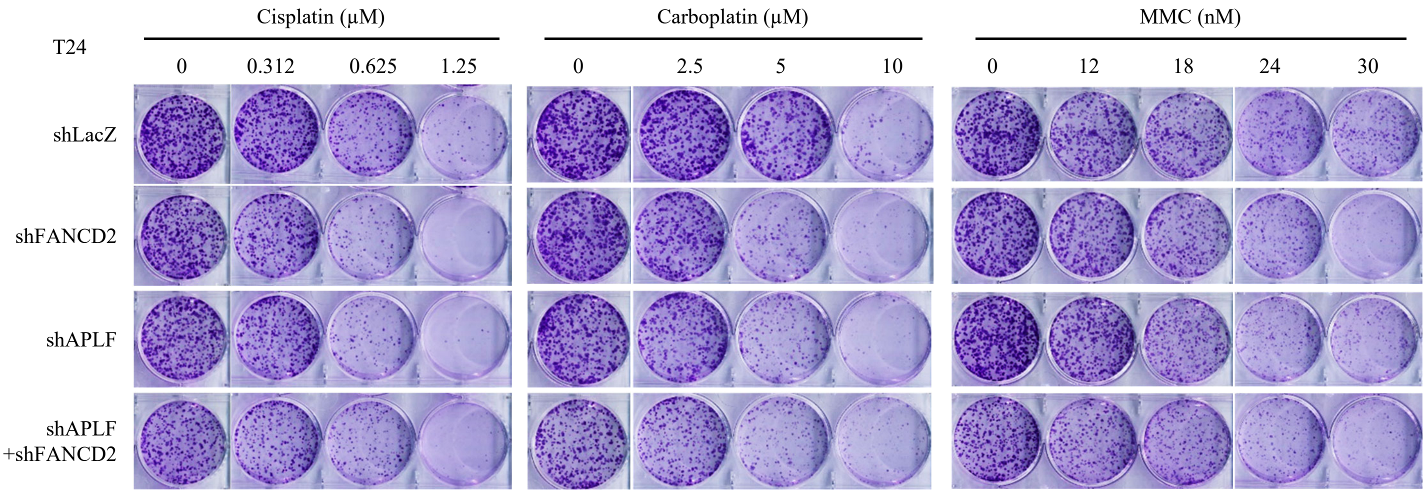

Figure S3. The single depletion of APLF or FANCD2 or double-depletion both of APLF and FANCD2 sensitizes cells to cisplatin, carboplatin, and MMC. Representative images of the colony formation assay. The expression of APLF and FANCD2 was depleted using shRNA lentivirus. These cells were chronically treated with various concentrations of cisplatin, carboplatin, or MMC for 10 days.

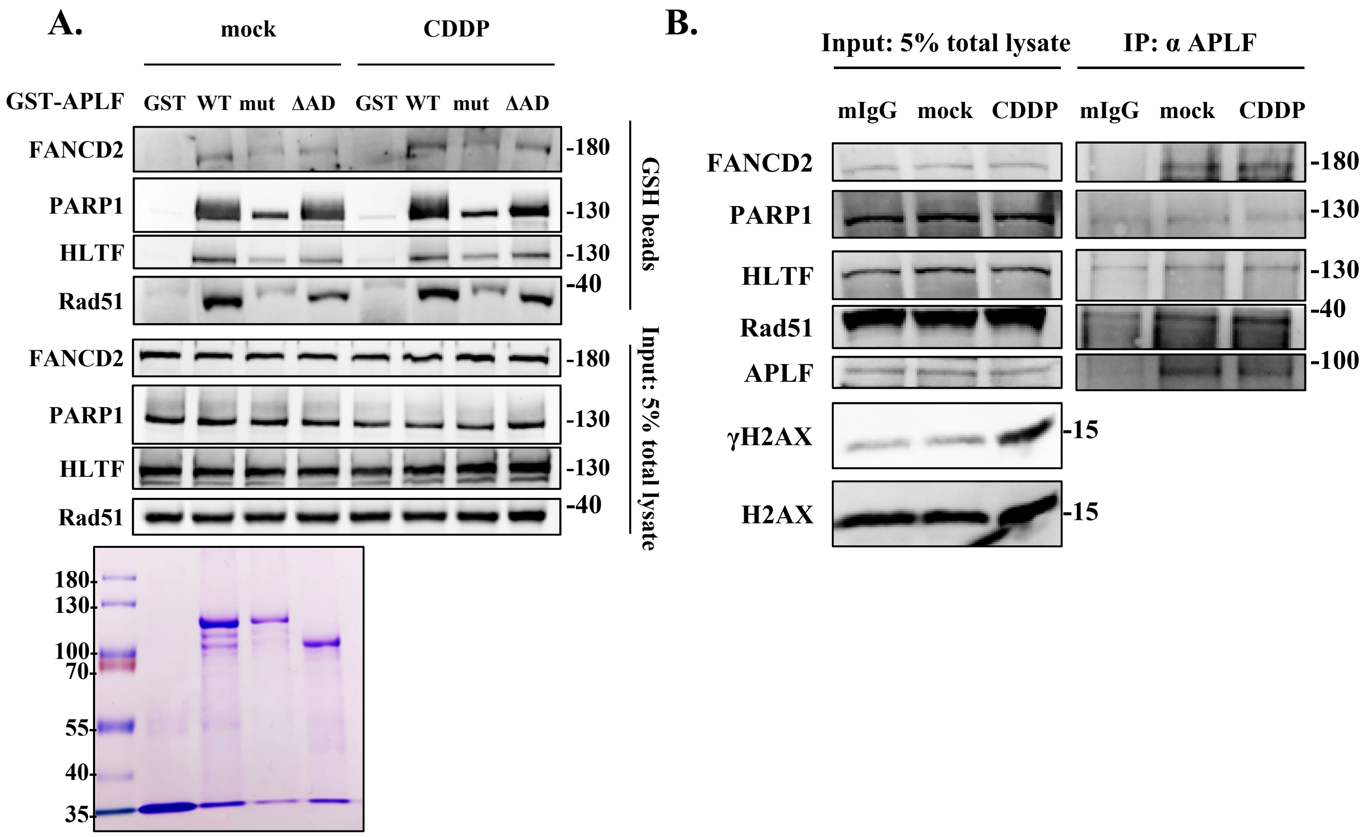

Figure S4. The PBZ mutant of APLF decreases its interaction with PARP1. (A) The GST pull-down assay. The GST-APLF fusion proteins were purified from *Escherichia coli Rosetta* (lower panel). HEK293T cells were treated with mock or 100 µM cisplatin for 3 hours. The cell lysates were incubated with each GST fusion protein. The proteins associated with each GST fusion protein were detected by antibodies as indicated. The GST alone (vector) was used as the negative control. (B) The coimmunoprecipitation assay. HEK293T cells were treated with mock or 100 µM cisplatin for 3 hr. The endogenous APLF was immunoprecipitated with an anti-APLF antibody and the proteins associated with APLF were analyzed by western blotting with specific antibodies as indicated. The non-specific mouse IgG (mIgG) was used as the negative control. Input represents 5% of total cell lysates.

A.

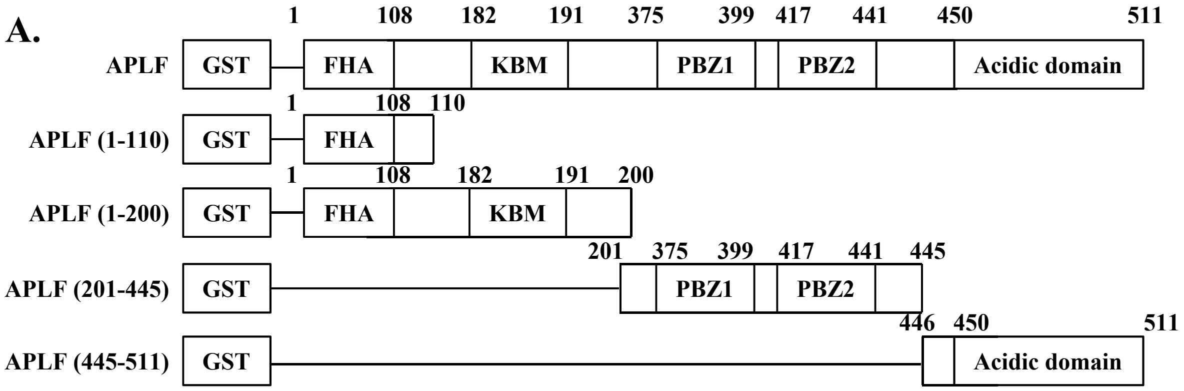

B.

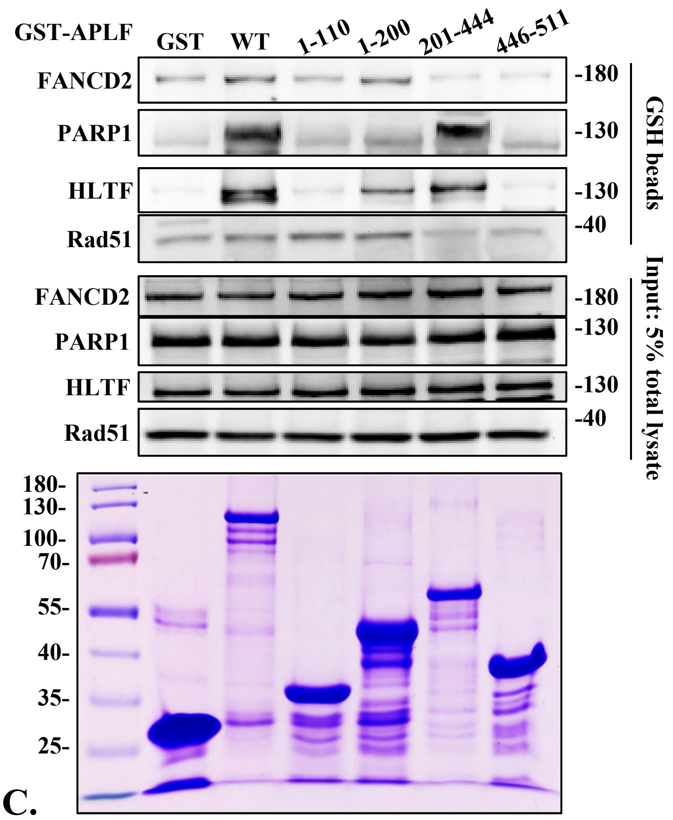

C.

Interactions	FANCD2	PARP1	HLTF	Rad51
APLF<br>Full length	+	+	+	+
APLF (1-110)<br>FHA domain	-	-	-	+
APLF (1-200)<br>FHA-KBM domain	+	-	+	+
APLF (201-445)<br>PBZ domain	-	+	+	-
APLF (445-511)<br>Acidic domain	-	-	-	-

Figure S5. The interaction domains of APLF with PARP1, HLTF, FANCD2, and RAD51.
(A) The schematic representation of APLF constructs. (B) These GST fusion proteins were purified from *E. Coli Rosetta* (lower panel). The cell lysates derived from HEK293T cells were incubated with each GST fusion protein. The proteins associated with each GST fusion protein were detected by antibodies as indicated. The GST alone (vector) was used as the negative control. (C) The interaction domains of APLF with PARP1, HLTF, FANCD2, and RAD51 are indicated as (+), while no interaction is indicated as (-).

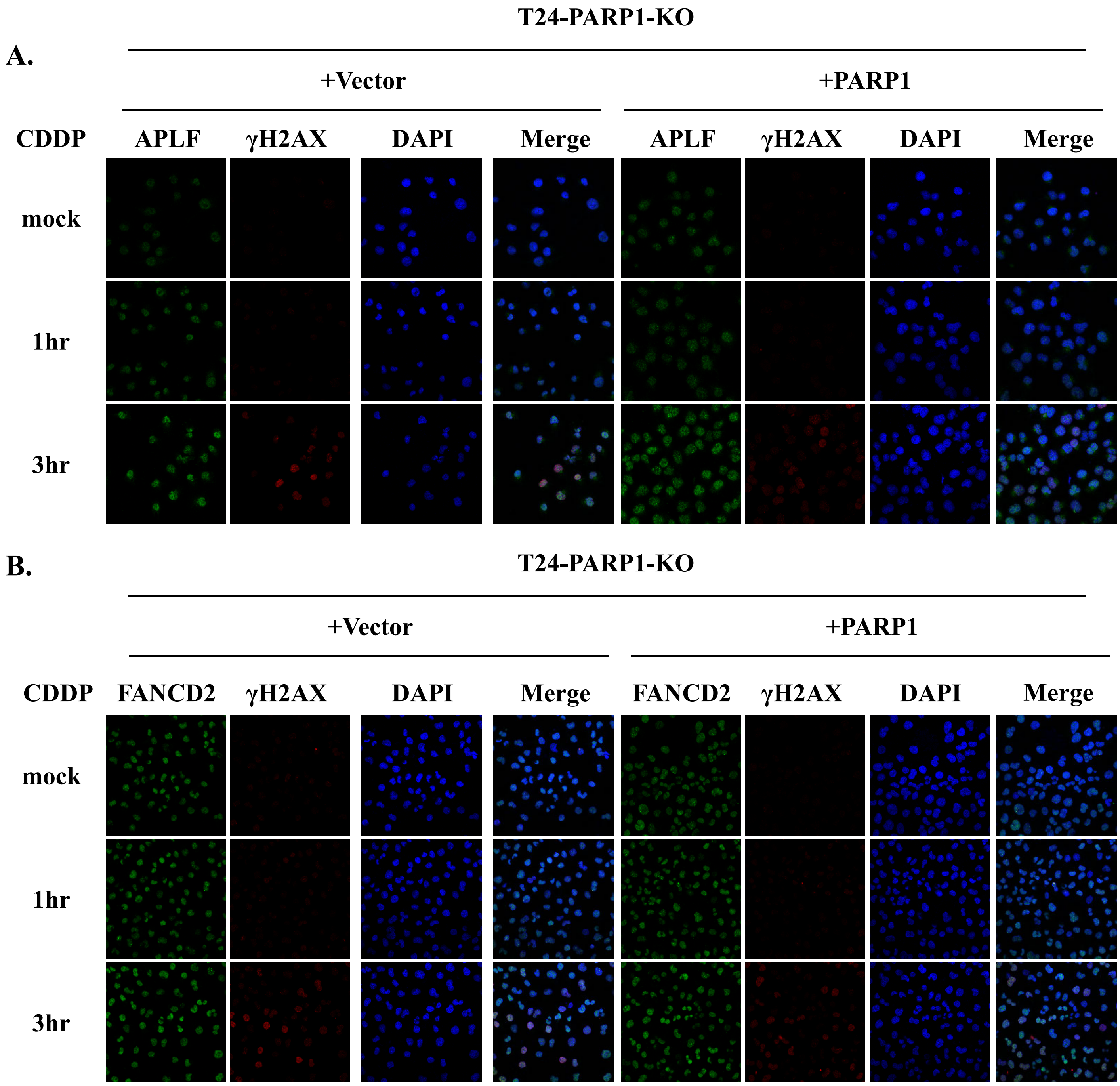
A.
T24-PARP1-KO
+Vector
+PARP1
CDDP
APLF
γH2AX
DAPI
Merge
APLF
γH2AX
DAPI
Merge
mock
1hr
3hr
B.
T24-PARP1-KO
+Vector
+PARP1
CDDP
FANCD2
γH2AX
DAPI
Merge
FANCD2
γH2AX
DAPI
Merge
mock
1hr
3hr

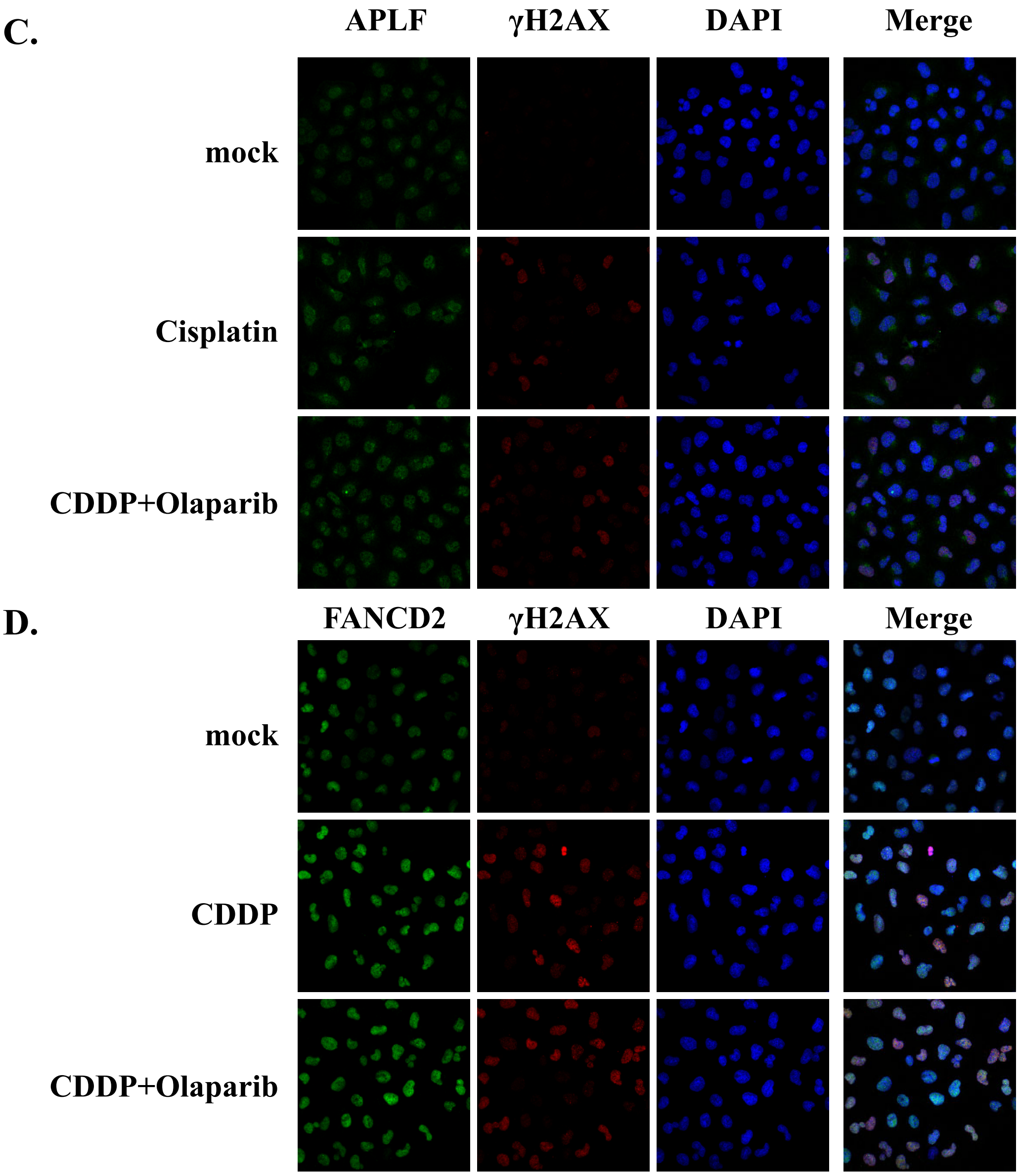

Figure S6. PARP1 facilitates APLF recruitment to DNA damage sites. (A) The representative images of APLF and γH2AX in PARP1-deficient (vector) and PARP1-proficient cells, following the treatment with mock or 100 μM cisplatin for 1 or 3 hr. The PARP1-KO (PARP1-deficient) T24 cells were infected with the retrovirus containing PARP1 to stably express wild-type PARP1. The empty vector was used as a control. (B) The representative images of FANCD2 and γH2AX in PARP1-deficient and proficient cells, following a similar treatment to (A). (C) The representative images of APLF and γH2AX in T24 cells with mock, cisplatin, or olaparib treatment. (D) The representative images of FANCD2 and γH2AX in T24 cells with mock, cisplatin, or olaparib treatment. All samples were acquired by Zeiss LSM780 Confocal Microscope and quantified by ZEN 3.6 (blue edition) software.

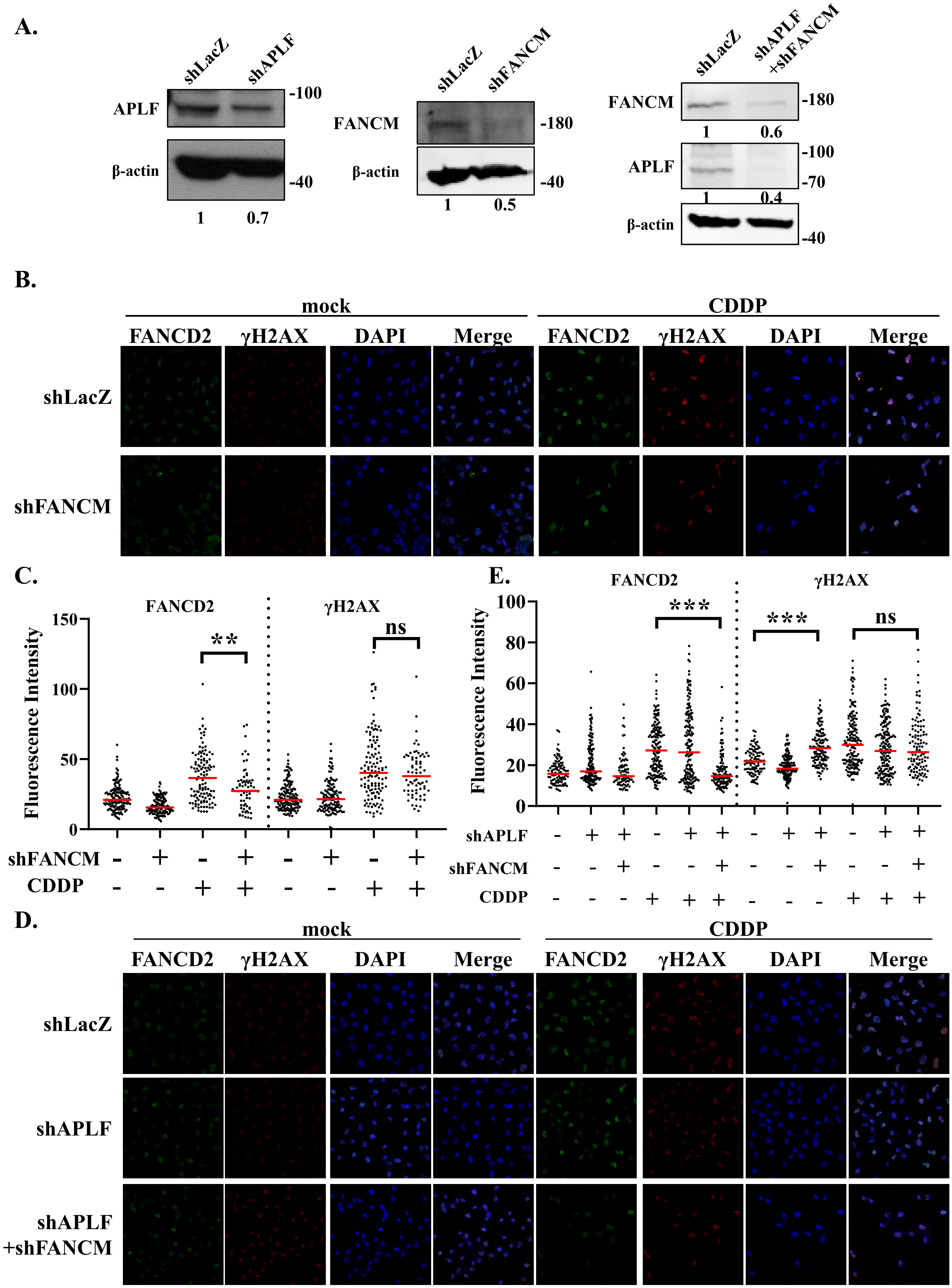

A.
shLacZ
shAPLF
APLF
-100
β-actin
-40
1
0.7
shLacZ
shFANCM
FANCM
-180
β-actin
-40
1
0.5
shLacZ
shAPLF +shFANCM
FANCM
-180
1
0.6
APLF
-100
-70
1
0.4
β-actin
-40
B.
mock
CDDP
FANCD2
γH2AX
DAPI
Merge
shLacZ
shFANCM
C.
FANCD2
γH2AX
Fluorescence Intensity
150
100
50
0
**
ns
shFANCM
CDDP
E.
FANCD2
γH2AX
Fluorescence Intensity
100
80
60
40
20
0
***
***
ns
shAPLF
shFANCM
CDDP
D.
mock
CDDP
FANCD2
γH2AX
DAPI
Merge
shLacZ
shAPLF
shAPLF +shFANCM

Figure S7. FANCM facilitates FANCD2 recruitment to DNA damage sites. (A) The depletion of APLF and FANCM was verified by western blot analysis with the specific antibodies as indicated. The expression of APLF and FANCM in T24 cells was depleted using shRNA-lentivirus. The non-targeting shLacZ was used as a control. (B) The representative images of FANCD2 and γH2AX staining in the shLacZ and shFANCM T24 cells. Cells were treated with mock or 100 μM cisplatin for 3 hr. (C) Quantification of FANCD2 and γH2AX fluorescent intensity after cisplatin treatment. At least 100 cells from each cell line were measured. (D) The representative images of FANCD2 and γH2AX staining in shLacZ, shAPLF, and shFANCM/shAPLF double-depleted T24 cells. Cells were treated with mock or 100 μM cisplatin for 3 hr. (E) Quantification of FANCD2 and γH2AX fluorescent intensity after cisplatin treatment. At least 100 cells from each cell line were measured. The *p-value* was determined by the Mann–Whitney test. ** represents $p < 0.01$; ***$p < 0.001$; ns, not significant. All samples were acquired by Zeiss LSM780 Confocal Microscope and quantified by ZEN 3.6 (blue edition) software.

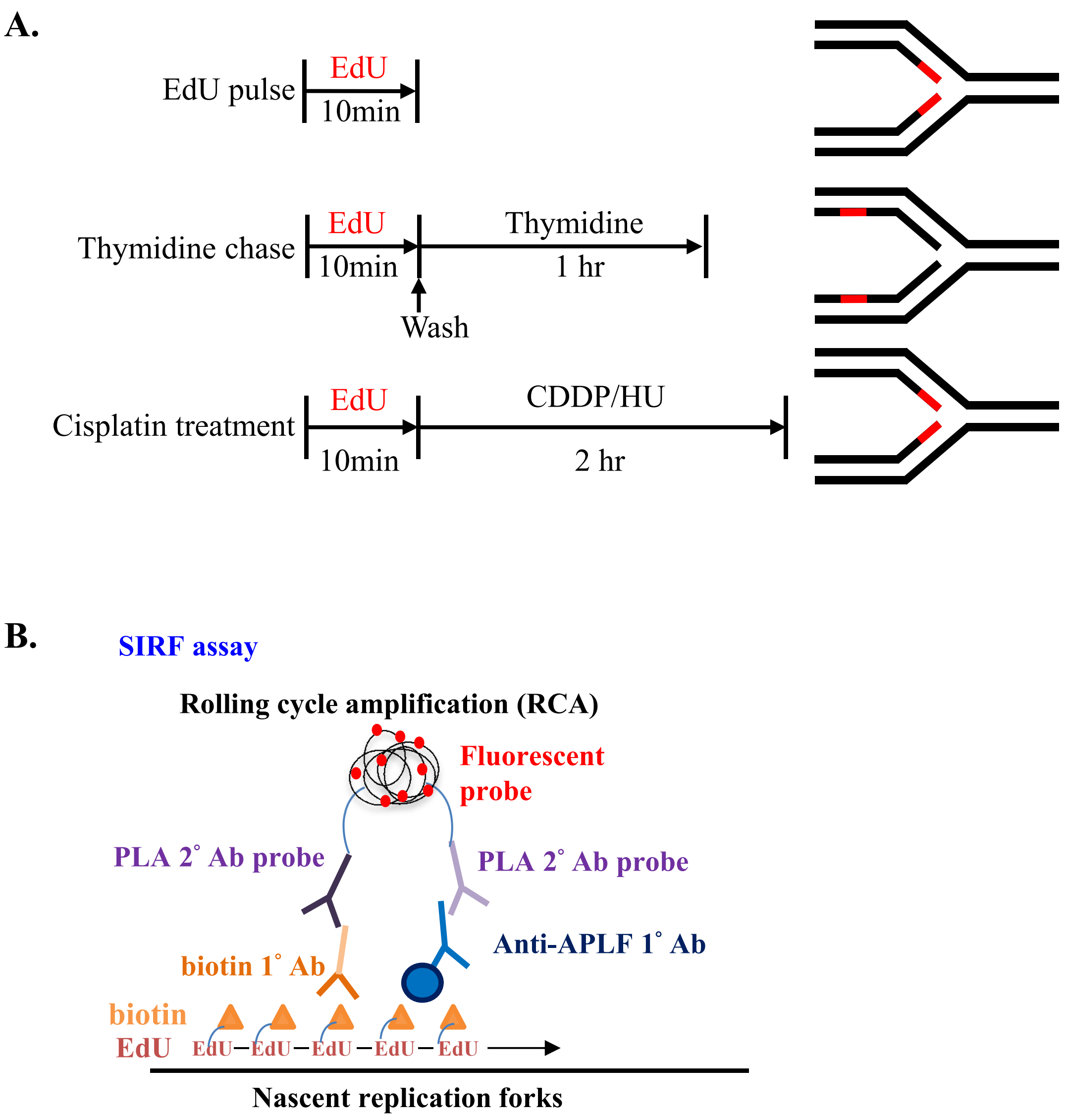

Figure S8. The schematic representation of iPOND and SIRF assays. (A) The schematic representation of the iPOND assay. The nascent DNA was pulse-labeled with EdU for 10 min, followed by a thymidine chase for 1 hr, 150 µM cisplatin or 4mM HU treatment for 2 hr. The corresponding replication forks are indicated on the right panel. (B) The schematic representation of the SIRF assay. The nascent DNA was pulse-labeled with EdU for 10 min. Following the click reaction, EdU was conjugated with biotin. The primary antibodies against biotin and the target protein (APLF) were used to detect nascent DNA and the target protein, respectively. Two oligonucleotide-conjugated secondary antibodies were used to bind to their primary antibodies. If the target protein was associated with EdU-labeled replication tracks, the oligonucleotides would hybridize to form circular DNA. Following rolling cycle amplification, the amplified DNA could be detected by a fluorescent probe, as revealed by the foci formation, referred to as the PLA foci.

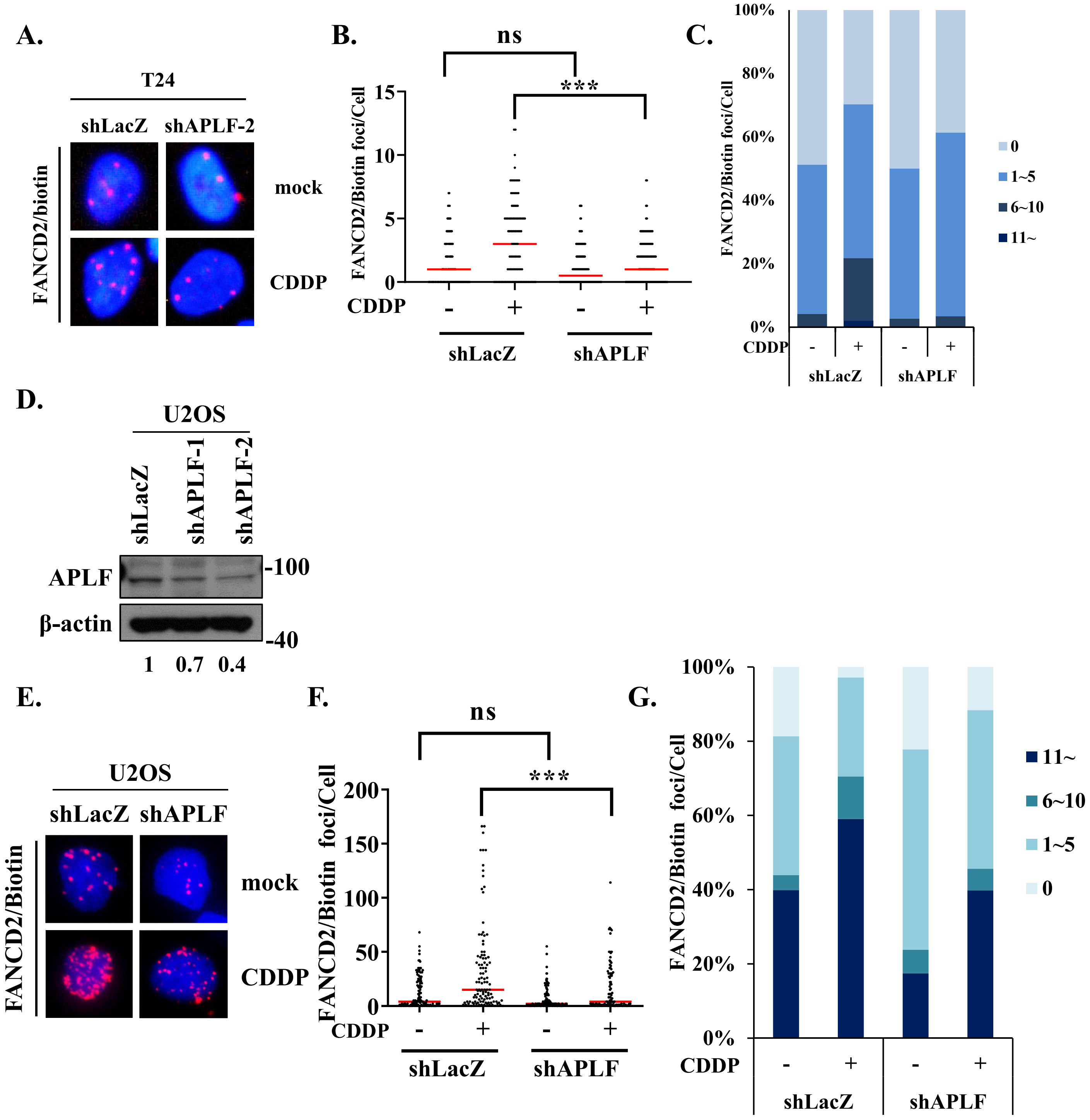

Figure S9. APLF depletion reduces the FANCD2 enrichment at stalled forks. (A) The representative images of FANCD2 SIRF assay in shLacZ and shAPLF-2 T24 cells. Cells were treated with mock or 100 µM cisplatin for 3 hr. (B) Distribution of FANCD2/biotin PLA foci from each cell line derived from (A). (C) The percent stacked column graph is derived from (B). (D) The immunostaining of APLF and FANCD2 from each cell line. The expression of APLF was depleted by shRNA-lentivirus. shLacZ was used as the control. (E) The representative images of FANCD2 SIRF assay in shLacZ and shAPLF U2OS cells. Cells were treated with mock or 100 µM cisplatin for 3 hr. (F) Distribution of FANCD2/biotin PLA foci from each cell line derived from (E). (G) The percent stacked column graph is derived from (F). At least 100 cells from each condition were measured. The *p-value* was determined by the Mann–Whitney test. ***$p < 0.001$; ns, not significant. All experiments have been repeated at least twice, with very similar results.

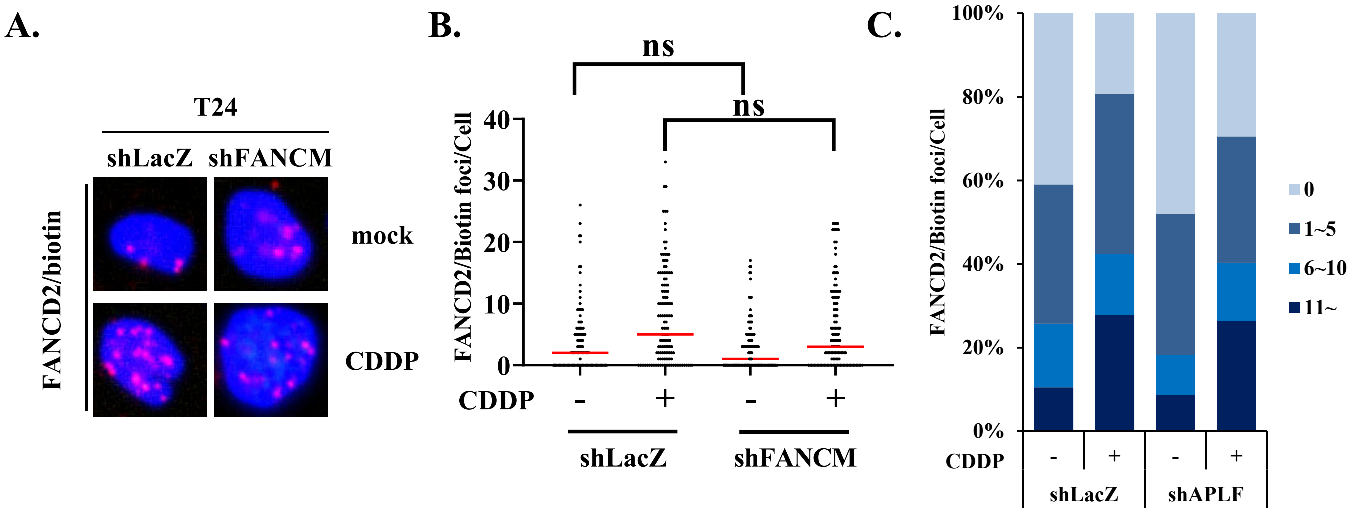

Figure S10. The FANCM depletion did not affect FANCD2 recruitment to stalled forks. (A) The representative images of FANCD2 SIRF assay in shLacZ and shFANCM T24 cells. Cells were treated with mock or 100 μM cisplatin for 3 hr. (B) Distribution of FANCD2/biotin PLA foci from each cell line derived from (A). (C) The percent stacked column graph is derived from (B). At least 100 cells from each condition were measured. The *p-value* was determined by the Mann–Whitney test. ns, not significant. All experiments have been repeated at least twice, with very similar results.

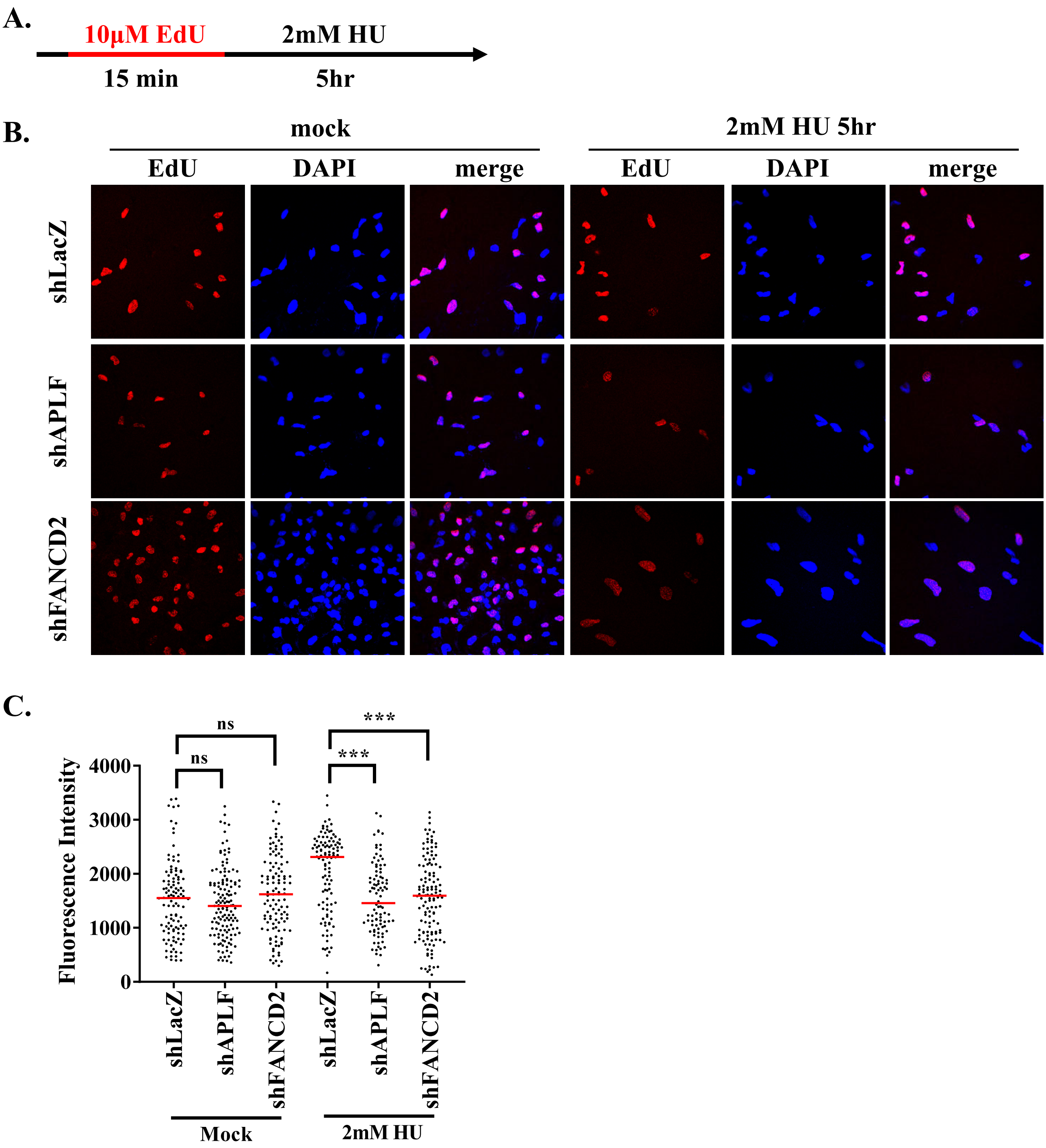

Figure S11. Depletion of APLF and FANCD2 significantly decreases EdU track length. (A) The labeling protocols for Cy5-fluorescent intensity analysis. (B) Representative images of Cy5-fluorescent intensity derived from each cell line. The images were acquired using a Zeiss LSM 780 confocal microscope. (C) Quantification of Cy5-fluorescent intensity. At least 200 cells from each cell line were measured in these experiments. The *p-value* was determined by the Mann–Whitney test. ***$p < 0.001$; ns, not significant.

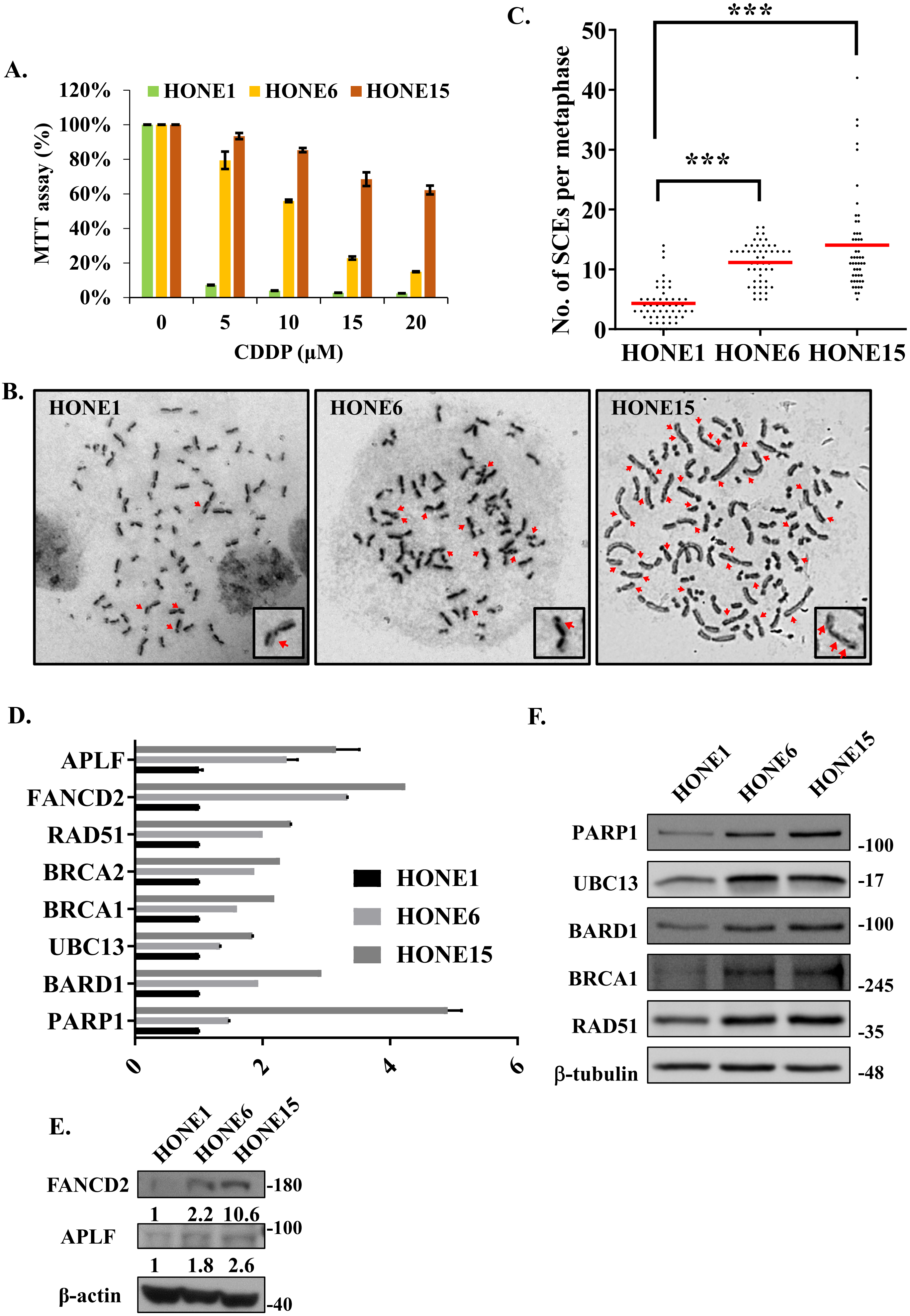

A.
120%
100%
80%
60%
40%
20%
0%
MTT assay (%)
HONE1
HONE6
HONE15
0
5
10
15
20
CDDP (μM)
C.
***
***
50
40
30
20
10
0
No. of SCEs per metaphase
HONE1
HONE6
HONE15
B.
HONE1
HONE6
HONE15
D.
APLF
FANCD2
RAD51
BRCA2
BRCA1
UBC13
BARD1
PARP1
HONE1
HONE6
HONE15
0
2
4
6
F.
HONE1
HONE6
HONE15
PARP1
-100
UBC13
-17
BARD1
-100
BRCA1
-245
RAD51
-35
β-tubulin
-48
E.
HONE1
HONE6
HONE15
FANCD2
-180
1
2.2
10.6
APLF
-100
1
1.8
2.6
β-actin
-40

Figure S12. APLF is highly expressed in cisplatin-resistant cells. (A) Cytotoxicity to cisplatin was determined by the MTT assay. HONE1, HONE6, and HONE15 cells were treated with cisplatin for 72 hr. (B) The SCE analysis of HONE1, HONE6, and HONE15 cells. SCE is indicated by arrows. (C) 50 metaphase cells from each cell line were measured. The *p-value* was determined by the Mann–Whitney test. ***$p < 0.001$. (D) Relative mRNA expression levels were determined by qRT-PCR and normalized by the levels in HONE1 cells. The expression levels of each gene were normalized by the levels of internal control ACTB. (E-F) The immunostaining of each gene is indicated.

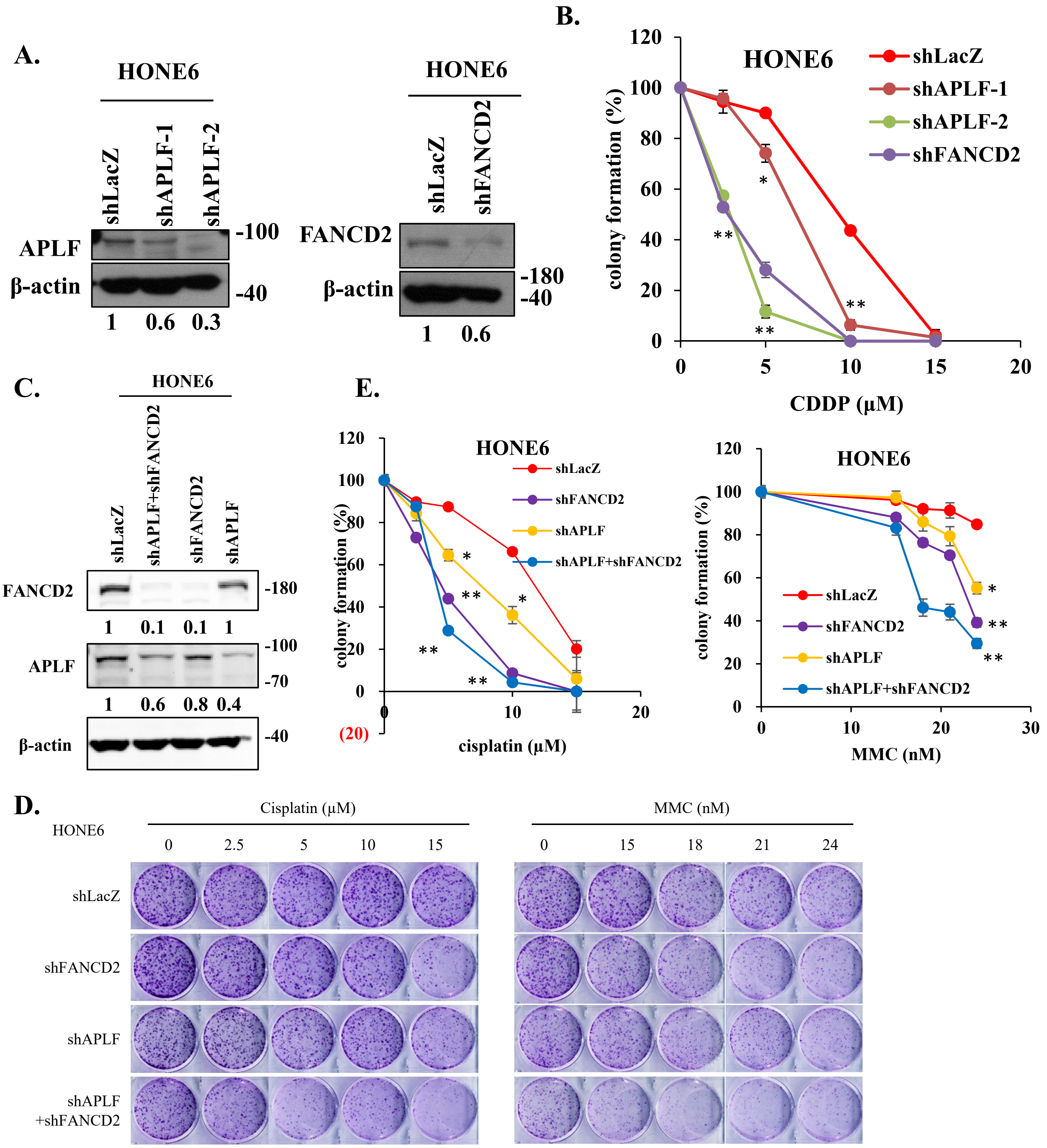

Figure S13. The depletion of APLF and FANCD2 sensitizes cisplatin-resistant cells to cisplatin and MMC. (A) The depletion of APLF and FANCD2 was verified by western blot analysis with the specific antibodies as indicated. The expression of APLF and FANCD2 was depleted using shRNA-lentivirus. The non-targeting shLacZ was used as a control. (B) The quantification of colony formation assay. Cells were chronically treated with cisplatin for 10 days. (C) The double-depletion of APLF and FANCD2 was verified by western blotting with the specific antibodies as indicated. (D) Representative images of the colony formation assay. HONE6 cells were chronically treated with various concentrations of cisplatin or MMC for 10 days. (E) The quantification of the colony formation assay. All data are the means ± standard deviation (SD) from at least two independent experiments. The *p-value* was determined by the student t-test. *$p < 0.05$; ** $p < 0.01$.

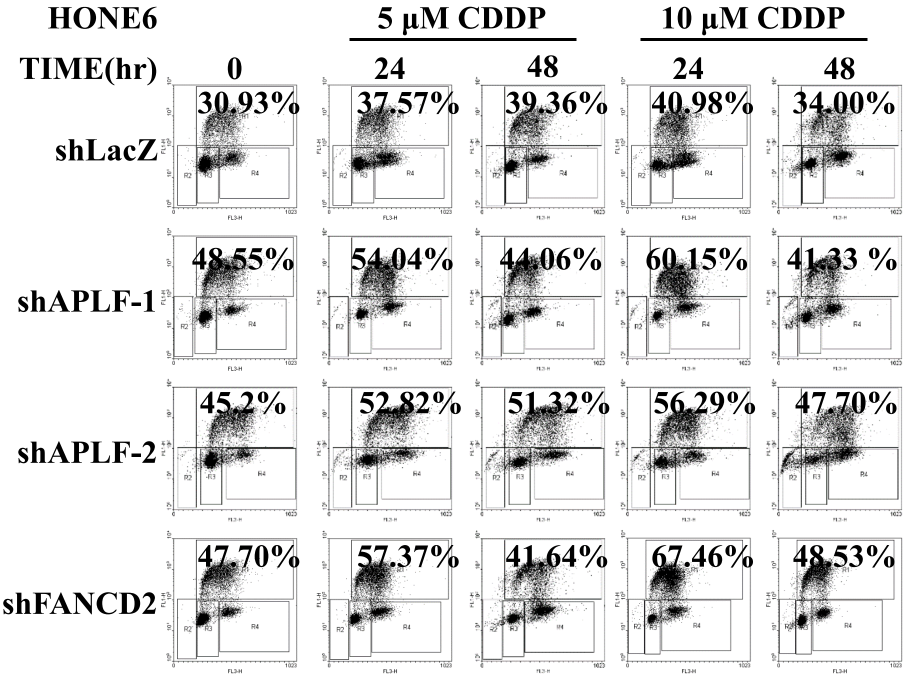

Figure S14. Depletion of APLF and FANCD2 accumulates cells in the S-phase of the cell cycle after cisplatin treatment. HONE6 cells were treated with 5 or 10 μM cisplatin for 24 or 48 hr. The cell cycle progress was determined by the flow cytometry.

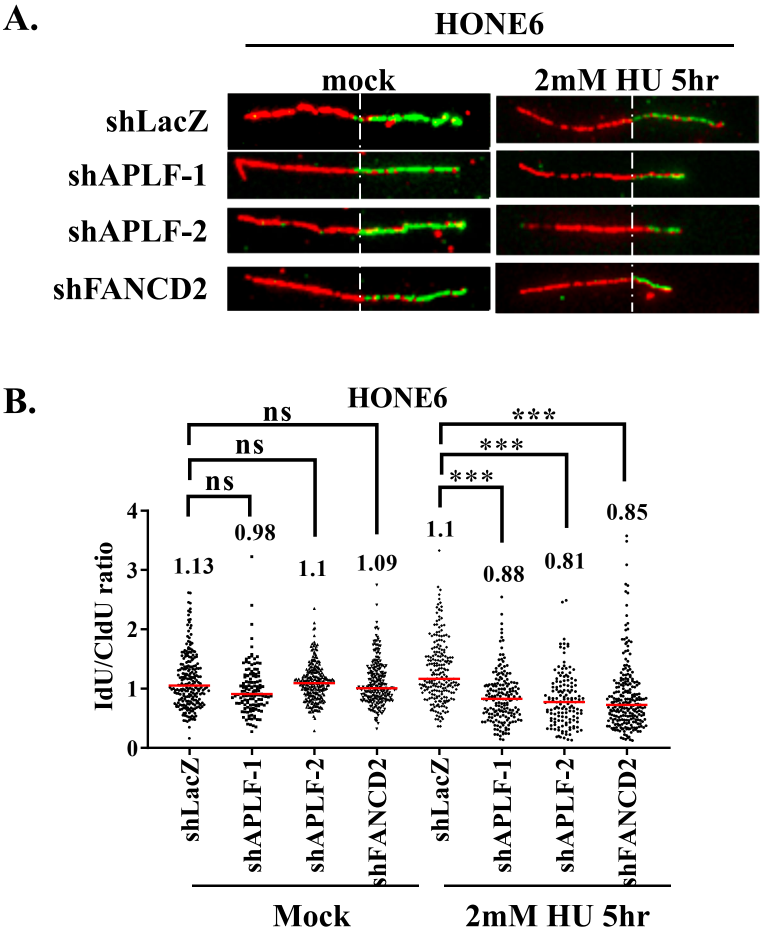

Figure S15. Depletion of APLF and FANCD2 significantly decreases IdU track length. (A) Representative images of DNA fiber analysis. (B) Quantification of IdU/CldU ratios from each cell line. At least 200 DNA fibers derived from each cell line were measured in these experiments. The *p-value* was determined by the Mann–Whitney test. ***$p < 0.001$; ns, not significant.

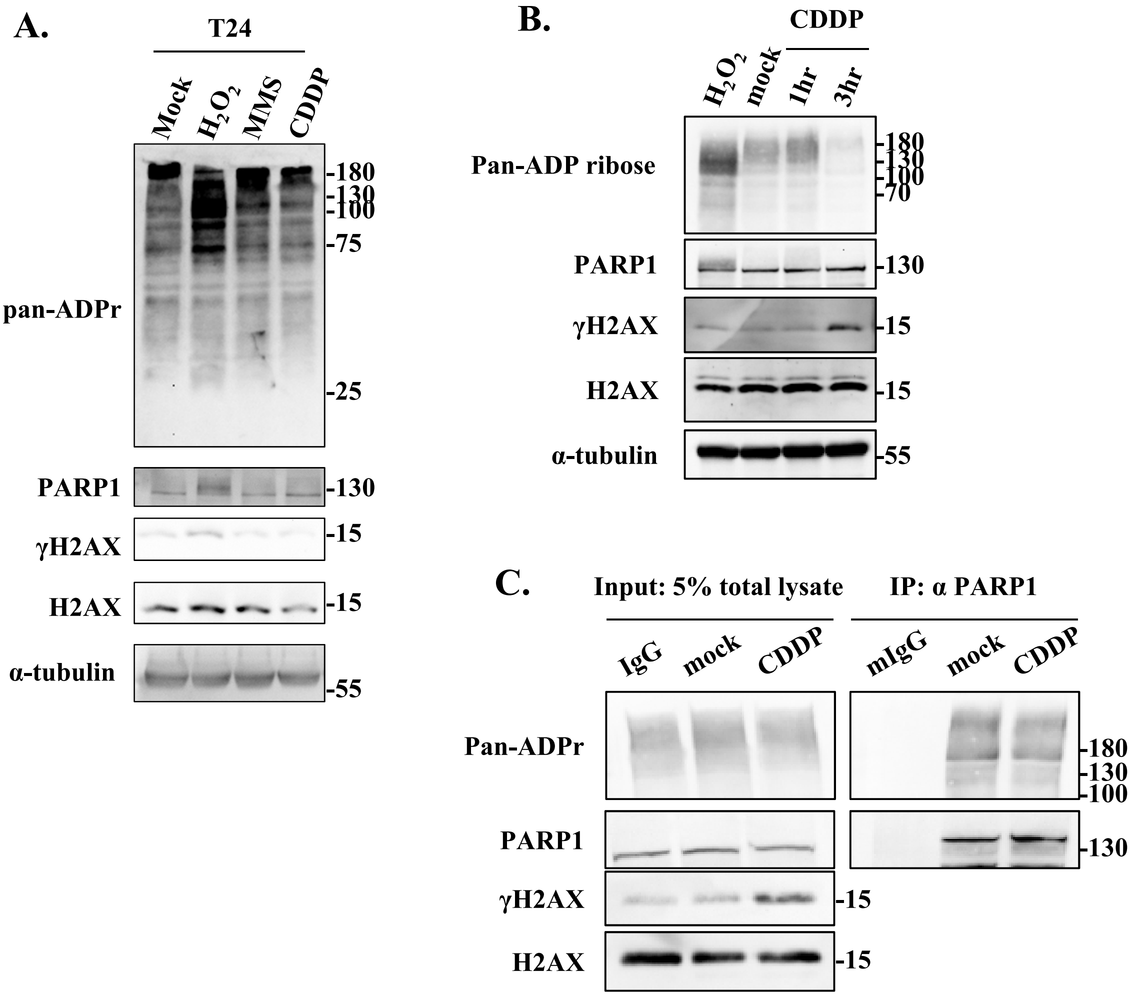

Figure S16. The pan-ADP-ribosylation has already occurred in the absence of DNA damage treatment. (A) T24 cells were treated with 1 mM H_2O_2 for 15 min, 1.2mM MMS for 15 min, or 100 μM cisplatin for 15 min. The total lysates were subjected to western blot analysis with the specific antibodies as indicated. (B) HEK293T cells were treated with 1 mM H_2O_2 for 15 min or 100 μM cisplatin for 1- or 3- hour. The total lysates were subjected to western blot analysis with the specific antibodies as indicated. (C) PARP1 was PARylated in the absence or presence of cisplatin treatment. HEK293T cells were treated with mock or 100 μM cisplatin for 3 hr. PARP1 was immunoprecipitated with an anti-PARP1 antibody and PARP1 PARylation was analyzed by western blotting with specific antibodies as indicated. Input represents 5% of total cell lysates.

Table S1. The list of antibodies used in this study.						
Antibodies	WB Dilute concentration	Confocal Dilute concentration	IHC Dilute concentration	PLA Dilute concentration	Catalog number	Source
Mouse monoclonal anti-PARP1	1: 2000				556494	BD Biosciences
Mouse monoclonal anti-PCNA	1: 2000			1:200	CBL407	Merck Millipore
Mouse monoclonal anti-beta-Actin	1: 10000				NB600-501	Novus Biologicals
Mouse monoclonal anti-a-tubulin	1: 10000				GTX628802	Gene Tex
Mouse monoclonal anti-BRCA1	1: 1000				ab16780	Abcam
Rabbit polyclonal anti-Rad51	1: 2000				ab63801	Abcam
Rabbit polyclonal anti-BARD1	1: 2000				A300-263A	Bethyl Laboratories
Rabbit polyclonal anti-UBC13	1: 1000				ab25885	Abcam
Rabbit polyclonal anti-HLTF	1: 1000				Cat#A300-230A	Bethyl Laboratories
Rabbit polyclonal anti-HA	1: 1000				H6908	Sigma-Aldrich
Rabbit polyclonal anti-H2AX	1: 1000				ab124781	Abcam
Mouse monoclonal anti-Flag	1: 1000				F1804	Sigma-Aldrich
Mouse monoclonal anti-γH2AX	1: 1000	1:200			Cat#05-636	Merck Millipore
Rabbit monoclonal anti-Histone H3	1: 5000				05-928	Merck Millipore
Rabbit polyclonal anti-APLF	1: 1000	1:200	1:100	1:100	GTX87979	Gene Tex
Rabbit polyclonal anti-FANCD2	1: 1000	1:200			ab2187	Abcam
Mouse polyclonal anti-turboGFP		1:200			TA150041	Origene
Rabbit monoclonal anti-γH2AX			1:100		9718	Cell Signaling Technology
Mouse monoclonal anti-biotin (PLA)				1: 2000	Cat#200-002- 211	Jackson ImmunoResearch
Rabbit polyclonal anti-biotin (PLA)				1: 2000	Cat#A150-109A	Bethyl Laboratories
Rabbit polyclonal anti-FANCD2				1: 200	PLA0243	Sigma-Aldrich
Rat monoclonal anti-BrdU	1: 200				ab6326	Abcam
Anti-pan-ADP-ribose binding reagent	1: 1000				MABE1016	Sigma-Aldrich
Rabbit polyclonal anti-FANCM	1: 500				HPA055144	Sigma-Aldrich
Rabbit polyclonal anti-PARP1		1:200			ab32138	Abcam
